# Qualichem *In Vivo*: A Tool for Assessing the Quality of *In Vivo* Studies and Its Application for Bisphenol A

**DOI:** 10.1371/journal.pone.0087738

**Published:** 2014-01-29

**Authors:** Laura Maxim, Jeroen P. van der Sluijs

**Affiliations:** 1 Institut des Sciences de la Communication du CNRS (UPS 3088), Centre National de la Recherche Scientifique, Paris, France; 2 Environmental Sciences, Copernicus Institute, Utrecht University, Utrecht, The Netherlands; University of Houston, United States of America

## Abstract

In regulatory toxicology, quality assessment of *in vivo* studies is a critical step for assessing chemical risks. It is crucial for preserving public health studies that are considered suitable for regulating chemicals are robust. Current procedures for conducting quality assessments in safety agencies are not structured, clear or consistent. This leaves room for criticism about lack of transparency, subjective influence and the potential for insufficient protection provided by resulting safety standards. We propose a tool called “Qualichem *in vivo*” that is designed to systematically and transparently assess the quality of *in vivo* studies used in chemical health risk assessment. We demonstrate its use here with 12 experts, using two controversial studies on Bisphenol A (BPA) that played an important role in BPA regulation in Europe. The results obtained with Qualichem contradict the quality assessments conducted by expert committees in safety agencies for both of these studies. Furthermore, they show that reliance on standardized guidelines to ensure scientific quality is only partially justified. Qualichem allows experts with different disciplinary backgrounds and professional experiences to express their individual and sometimes divergent views—an improvement over the current way of dealing with minority opinions. It provides a transparent framework for expressing an aggregated, multi-expert level of confidence in a study, and allows a simple graphical representation of how well the study integrates the best available scientific knowledge. Qualichem can be used to compare assessments of the same study by different health agencies, increasing transparency and trust in the work of expert committees. In addition, it may be used in systematic evaluation of *in vivo* studies submitted by industry in the dossiers that are required for compliance with the REACH Regulation. Qualichem provides a balanced, common framework for assessing the quality of studies that may or may not be following standardized guidelines.

## Introduction

Biomedical research has been evaluated using quality assessment frameworks for many years. Existing scales use between 2 and 100 criteria to assess the methodological quality of clinical trials [1]. For example, the Jadad scale provides a 7-point checklist for assessing the quality of clinical trials in pain research [2]. The proposed GRADE (Grading of Recommendations, Assessment, Development, and Evaluation) framework evaluates the quality of evidence and strength of recommendations about therapeutic and diagnostic interventions and clinical management strategies [3]–[4]. Adapted to the Australian context, the FORM framework was created to formulate and grade recommendations in clinical practice guidelines [5].

Transparent and complete reporting is key for assessing the methodological quality of a study. Therefore, there are specific recommendations for reporting (for example) randomized controlled trials [6] and observational studies in epidemiology [7].

Quality assessment frameworks for academic and regulatory toxicology are less developed. Wandall et al. [8] made one of the first attempts to identify sources of bias in toxicology, but did not develop a quality assessment framework. Highlighting the importance of adequate reporting for informing policy and scientific practice in animal research, Kilkenny et al. [9] proposed the ARRIVE (Animals in Research: Reporting *In vivo* Experiments) guideline for reporting *in vivo* studies. The objective of this guideline is to allow in-depth critique of reported quality controls using a framework for including all relevant information about what was done, why and how. However, very few complete frameworks for quality assessment of *in vivo* toxicology studies have been proposed and/or tested. Among them, the Klimisch score [10] defines data quality by three properties: adequacy, relevance and reliability.

Public health in Europe depends critically on effective implementation of the REACH (Registration, Evaluation, Authorisation & restriction of CHemicals) regulation, which concerns the risks of most chemicals on the market. Protection of public health will not be effective without ensuring the data submitted by industry—on which political decisions are based—is of high scientific quality. REACH uses the Klimisch score [11] to assess the quality of individual studies. However, the method used to assess a study's adequacy and relevance leaves significant room for subjectivity. It is relatively easy to assess reliability of data arising from standardized tests—in particular OECD or national guidelines and good laboratory practice (GLP). However, the definition of reliability in the Klimisch score disadvantages studies that do not follow standardized guidelines, but are nevertheless scientifically robust; i.e., most of the published academic literature. In REACH, the Klimisch score is often applied by industry itself, when submitting data to health agencies. Industry must assess its own studies or studies from the academic literature. Room for subjectivity in assessing studies may lead to selection bias in choosing and weighting the set of studies that is ultimately used by industry and health agencies to inform decision-making.

Recognizing the lack of precision of the Klimisch categories and the need for a more transparent, harmonized and objective framework for assessing the reliability of the toxicological data submitted under REACH, the ToxRTool was created [12]. However, criteria included in frameworks such as ToxRTool or ARRIVE focus on how completely a study is reported rather than on its scientific quality. But, reporting of seemingly straightforward details such as the study species or strain chosen can be a source of scientific debate about, for example, the sensitivity of that species or strain to estrogens [13].

Existing tools fail to provide a systematic approach for assessing the quality of *in vivo* studies used to inform policy by institutions charged with implementing regulatory frameworks or responding to requests for policy advice. Examples of such institutions relevant to chemical risks are EFSA (European Food Safety Authority) and ECHA (European Chemicals Agency).

Our paper aims to fill this methodological gap by developing and testing the Qualichem *in vivo* tool (or simply, “Qualichem”). For endocrine disrupters in general and BPA in particular, the challenge is to incorporate divergent views from scientists with affiliations in industry, academia, health agencies at the national and European level, and governments by providing a synthesized view of the global quality of a study. Previous tools like ToxRTool assume that heterogeneity in ratings is not a natural consequence of the differences among respondents (discipline, level of competence on the subject, previous experience, epistemic communities, etc), and that it can be solved if the questions are framed better. This assumption does not reflect real life situations: when evaluating studies of controversial topics like endocrine disrupters, scientists from different disciplinary backgrounds and socio-economic horizons openly disagree. Literature shows that the same raw data can be interpreted differently by different experts in different contexts, which can lead to conflicting conclusions [14]. Our approach solves the problem of ToxRTool's unrealistic assumption, as it incorporates the differences among respondents, and allows for a useful representation of the entire range of responses.

Furthermore, Qualichem could be used to include a wider range of studies in risk assessment in a more balanced way. Quality assessment using Qualichem would apply the same criteria to evaluate both industry studies that follow OECD or GLP guidelines and non-standardized academic studies that also provide scientific knowledge that is useful for decision-making.

As industrial chemicals such as BPA are present in many consumer products [15], studies used to create a regulatory framework have the potential to impact the lives of millions of people; as such, assessments of them must be rigorous. Quality assessment is a key step in helping to choose which studies regulatory decisions should be based on.

The role of regulatory science is to provide the best available scientific knowledge at a certain moment, and not the unachievable ideal of “perfect” knowledge. In line with the post-normal science proposal for addressing the robustness of science used to set policy [16], the Qualichem tool addresses more than lack of knowledge (epistemological uncertainty), and looks more broadly at the concept of quality, which also includes the following dimensions:

- **technical:** incorporates technical errors caused by imprecise instruments or measurement methods.
- **methodological:** incorporates whether and how researchers use the best available scientific knowledge and practices in drafting the research protocol, make assumptions when knowledge is lacking or choose among several available methods for assessing a parameter.
- **normative:** incorporates interpretation of raw data and conclusions about the level of evidence provided by that data.
- **communication:** incorporates how completely and understandably the research is reported.

Compared with the current framework for evaluation that is commonly used in regulatory chemical risk assessment [11], our definition of quality covers both *relevance* and *reliability*. In addition, quality includes aspects related to the interpretation and communication of the results, and to technical aspects of measurement, e.g., analytical techniques. Most importantly, the concept of quality highlights the importance of the knowledge production process—which directly influences the robustness and usefulness of scientific results used for a particular decision-making situation—instead of focusing on the results alone. Our approach aligns with previous work on knowledge quality assessment (KQA) tools, which are essential for timely and adequate policy responses in situations of risk governance [17] and for responding to the credibility crisis of science used to set controversial policy [18]–[19]. We draw on previous experience with the KQA tool NUSAP (Numeral, Unit, Spread, Assessment, Pedigree) [20], already used to assess the quality of estimates of NO_x_, SO_2_, NH_3_ [21] and volatile organic compound emissions [22] in the Netherlands and to assess health risks from tropospheric ozone [23] emissions from a waste incinerator [24]; and from electromagnetic fields from overhead power lines [25].

The objective of Qualichem is to provide a systematic and transparent framework to assess the quality of studies used in regulatory chemical risk assessment (Materials and methods). To validate this tool, it was tested with relevant academic and health agency scientists, and its applicability checked using both short (several pages) and long (4,000 pages) studies (Results). Other objectives of this paper are to 1) compare the quality criteria addressed in our tool with those previously used by European institutions that provide expertise on the risk of BPA and by the OECD and OPPTS standardized guidelines relevant to the two BPA studies evaluated here (sections 3.2. and 3.3.), 2) examine whether some criteria hold more weight in determining the final quality of a study (section 3.4), and 3) examine whether quality assessments are influenced by the disciplinary background and publication history of the respondents (section 3.5).

## Materials and Methods

### Ethics statement

This study did not involve patients, and written consent was not required. Consent to participate was voluntary and was obtained by email. Anonymity and confidentiality of the interviews were guaranteed to all participants. The interview protocol has been sent to participants before the meeting. The participant was then asked to give oral consent and to allow audio recording of the interview. We did conduct research outside our country of residence but approaching local authorities was not needed because interviewees' institutional information were not used for our project. The ethics evaluation committee of Inserm (IORG0003254, FWA00005831), the Institutional Review Board (IRB00003888) of the French Institute of medical research and Health, approved the study protocol, including the information sheet on the expert profile and the oral consent procedure (Opinion number 13-123).

### 2.1. The quality criteria: an original typology

We developed the typology of quality criteria (Text S1) iteratively, following the main steps of the process of knowledge production of *in vivo* studies; using ECHA's guidelines for the evaluation of information [11]; analysis of study criticism expressed by scientists (e.g., [13], [26]) or safety agencies like EFSA; previous literature on reporting *in vivo* studies [9], [12] and on sources that look at heterogeneity in expert judgments [8], [14], authors' personal experiences with regulatory documents and authors' expertise in a safety agency. In these sources, we identified the criteria used to criticize, argue in favor of, or evaluate the scientific robustness of *in vivo* studies. We considered the various lines of argumentation identified as expressions of expert judgments about *in vivo* studies, and that were therefore relevant criteria to include in our typology.

To check the robustness of our typology and incorporate feedback from the scientists interviewed, our interview protocol contained a final question about the need to exclude criteria or to include new ones.

We tested the typology with 12 scientists in academia and health agencies—a sample that is in line with the current literature on expert elicitation [27] that recommends 6 to 12 experts. A thirteenth expert validated the typology but his responses have been excluded from the Qualichem analysis. He only had time to give a general assessment of the study and did not use the proposed Likert scale. Due to lack of time, two of the twelve experts responded only to the questions referring to the criteria in the “Protocol” part of our typology (Table 1, Text S1). We used two case studies—a journal article (Tyl et al., 2002) [28] and a 4,000-page report (Stump, 2009) [29]—to test if our protocol can be used within a reasonable time frame on both short and longer studies. Both studies were funded by the chemical industry, which is common in regulatory assessment of chemical risks.

**Table 1:** Typology of quality criteria for *in vivo* studies.

Class	Quality criteria
Protocol Quality Criteria	
1. Substance	Check of substance properties; check of storage conditions; procedure for obtaining formulations; choice of the control
2. Experimental animals	Correspondence between the characteristics of tested animals and the characteristics of exposed humans; choice of test species/strain; handling of experimental animals; monitoring of experimental animals; monitoring of controls
3. Assay	Sensitivity of the assay; choice of experimental unit; number of groups tested; number of control groups; robustness of regulatory guidelines; test of a single substance or mixture
4. Measured effects	Parameters observed; observation time; biological level observed; precision of effects measurement
5. Tested exposure	Toxicokinetic stage for measuring exposure; level of doses tested; exposure duration; number exposure levels; route of administration; precision of exposure measurement; control of confounders
6. Laboratory procedures and human factors	Experimenter bias
Results Quality Criteria	
7. Results reporting	Results reporting; graphical data representation; abstract vs. raw data
8. Results analysis	Statistical methods used; statistical unit; treatment of data for statistics; statistical power; evaluation of errors, uncertainty, variability
9. Causal interpretation	Interpretation of dose-response; biological mechanism; extrapolation from animals to humans; functional relevance of changes
10. Results interpretation: epistemological context	Epistemological background
11. Results check	Status of peer-review; coherence with literature
12. Results interpretation: expert judgment	Results vs. raw data; assumptions
13. Variability	Variability

Our 45 quality criteria (defined in Text S2) are assembled into thirteen different classes (Table 1) that fall into two general categories: “Protocol” and “Results”. The Protocol part of the typology includes quality criteria that are relevant to the technical and methodological aspects of best available scientific knowledge and practices. The Results part includes only two criteria for technical and methodological quality: the results analysis and results check. The remaining results criteria pertain to communicational quality (such as results reporting) and normative quality (such as causal interpretation, interpretation in light of the existing epistemological background, and expert judgment of the level of evidence provided by the results) (Text S1).

The number of criteria evolved slightly thorough the interviews, based on comments from the experts. Therefore, some experts did not use the full set of 45 criteria. Four of the 45 criteria were added after interviews with two experts, one more criterion was added after the interview with the third expert, and two additional criteria were added after interviews with the sixth expert. The six remaining experts used the full set of 45 criteria and considered it to be complete.

### 2.2. Elicitation protocol

We interviewed each expert respondent individually in either 2012 or 2013. To prepare respondents for the interviews, we pasted relevant text from the study below each question. This saved respondents from having to search the study for the elements needed to answer the question or from using their memory to recall the relevant information, which could lead to imprecise responses.

Both studies claimed to comply with regulatory guidelines, and details of the guidelines that were relevant for assessing the study may not have been reported as part of the study. For this reason, we also copied the elements of the guidelines appropriate to each criterion in the survey.

Each respondent assessed one of the two studies, not both. To assess the quality of each study—specifically, how well they incorporate best scientific knowledge and practices—we presented each respondent with a question related to each of the criteria included in our typology. Elicitation protocols can be provided on demand. For example, the first question of our protocol was: “*Were the substance's properties checked before and during the experiment, in accordance with best scientific practices?*” The text from the study that refers to the check of substance properties was copied below the question. The respondent was invited to answer using a Likert scale (Table 2) and to explain his/her response (Text S7).

**Table 2:** Scale used for expert elicitation.

Answer	On a scale from 1 to 6, the answer corresponds to the score
Agree strongly	6
Agree moderately	5
Agree slightly	4
Disagree slightly	3
Disagree moderately	2
Disagree strongly	1
I cannot answer	CA
Not applicable	NA

Interviews were recorded and transcribed. We used the transcriptions to analyze the results (Results section, Text S3 and Text S4).

### 2.3. Choice of respondents

Respondents were either chosen through an extensive search of international peer-reviewed literature for authors of articles on BPA toxicology, were experts who had participated in BPA working groups in health agencies in Europe or were specialists in BPA and/or endocrine disrupters with expertise relevant to health agencies that were recommended by the scientists involved in our project. We searched all personal and other web pages and then listed disciplinary areas using the exact wording found in these documents, without trying to create exclusive classes. As a result, some disciplinary areas on our list overlap and some encompass others.

Following this process, we contacted 64 scientists by email. Thirteen agreed to participate. Four respondents were employed by safety agencies and nine were academics.

### 2.4. Choice of case studies

The controversy over the health risks of BPA repeatedly focuses on the quality assessment methods used in different health agencies, and on the reliance on standardized guidelines rather than academic research to select pivotal studies.

The two studies used as case studies here played an important role in BPA regulation. Tyl et al. (2002) was used as a critical study for choosing the NOAEL for BPA in Europe. Stump (2009) was devised in response to divergent views about BPA neurotoxicity between three Nordic and other European countries; it has been extensively reviewed by an EFSA working group. Tyl et al. (2002) has been considered robust enough to drive regulatory decisions [30]–[31], while Stump (2009) has been considered invalid [32]–[33].

### 2.5. Definition of controversial criteria: a measure of aggregated quality

It can be cumbersome and difficult to read a graphical representation of 45 criteria. To facilitate understanding and focus on the most important results, we have defined two categories of criteria: controversial and critical. The subset of criteria that we call controversial or critical is specific to each study assessed with Qualichem; they are not pre-defined as such, but are based on the outcome of the evaluation.

The two categories of criteria, controversial and critical, allow us to distinguish two levels of quality:

- **aggregated quality for each criterion**, using the median of the expert respondents' scores; this is an indicator of majority (consensus) views on aspects of study quality.
- **level of confidence** for the whole study, using a decision rule based on critical criteria (see below); this is an indicator of divergence between expert respondents, and gives important weight to the scores of critical expert respondents.

**Controversial criteria** are those for which, on the scale from 1 to 6 (Table 2):

- at least one respondent gave a score of 3 or less, or
- there is a difference of at least two points between any two scores.

The graphical representation shows all controversial criteria. All the other criteria—i.e., those that are not controversial according to our definition—received scores of 5 or 6 and were considered to have a high aggregated quality.

The graphical representation was built using a tailored Excel file (Text S8, Text S9, Text S10). The graphic is divided into three colored areas: red (including scores and median scores <3), orange (for scores and median scores between 3 and 4) and green (for scores and median scores >4). For each criterion, a line covers the full range, from the lowest score to the highest score in the group of responding experts. The median score is represented by an “x” and the interquartile range is represented by a rectangle.

**The aggregated quality of an individual criterion** is assigned as follows:

- **High aggregated quality**: median in the green area (>4)
- **Average aggregated quality**: median in the orange area (ranging from 3 to 4)
- **Low aggregated quality**: median in the red area (<3).

**Figure 1:**
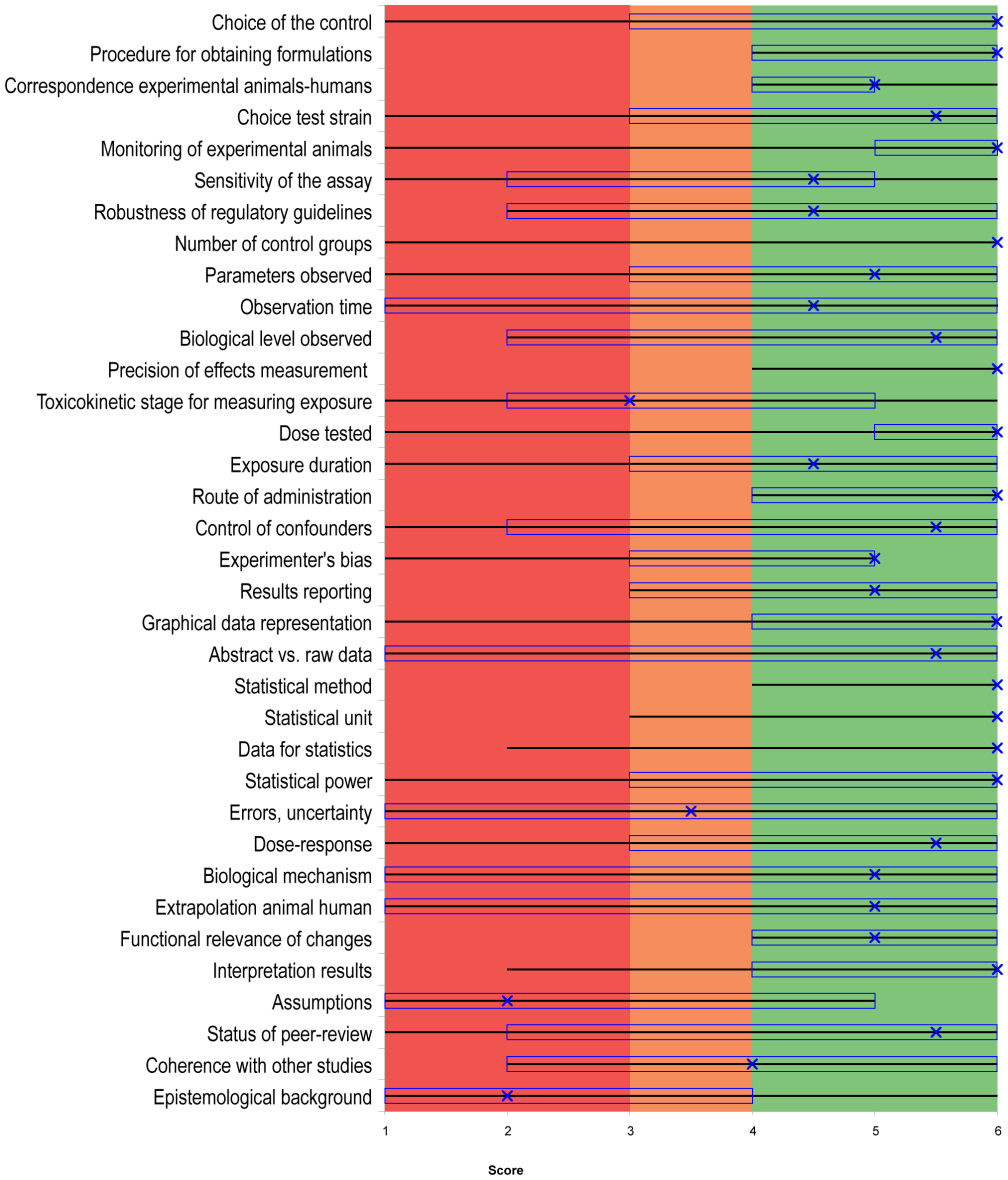
Quality assessment of Tyl et al. (2002), using Qualichem with eight respondents. For the study of Tyl et al. (2002), of the 45 criteria, the figure represents only the 35 controversial criteria out of the total set of 45 criteria. The remaining 10 criteria were not controversial according to our definition; they received scores of 5 or 6 and were considered to be of high aggregated quality. The figure is divided in three colored areas: red (including scores and medians <3), orange (for scores and medians between 3 and 4) and green (for scores and medians >4). A line covers the full range, from the lowest score to the highest score in the group of responding experts. The median of the scores is represented by an “x” and the interquartile range is represented by a rectangle. If the median (x) is in the red area, the aggregated quality of the criterion is low. If the median is in the orange area, the aggregated quality is average. If the median is in the green area, the aggregated quality is high. The interquartile range is an indicator of inter-expert heterogeneity. Thirty of the 35 controversial criteria were of high aggregated quality (the median is in the green area). Of the five remaining criteria, three were of average aggregated quality (the median in the orange area) and two were of low aggregated quality (the median is in the red area).

**Figure 2:**
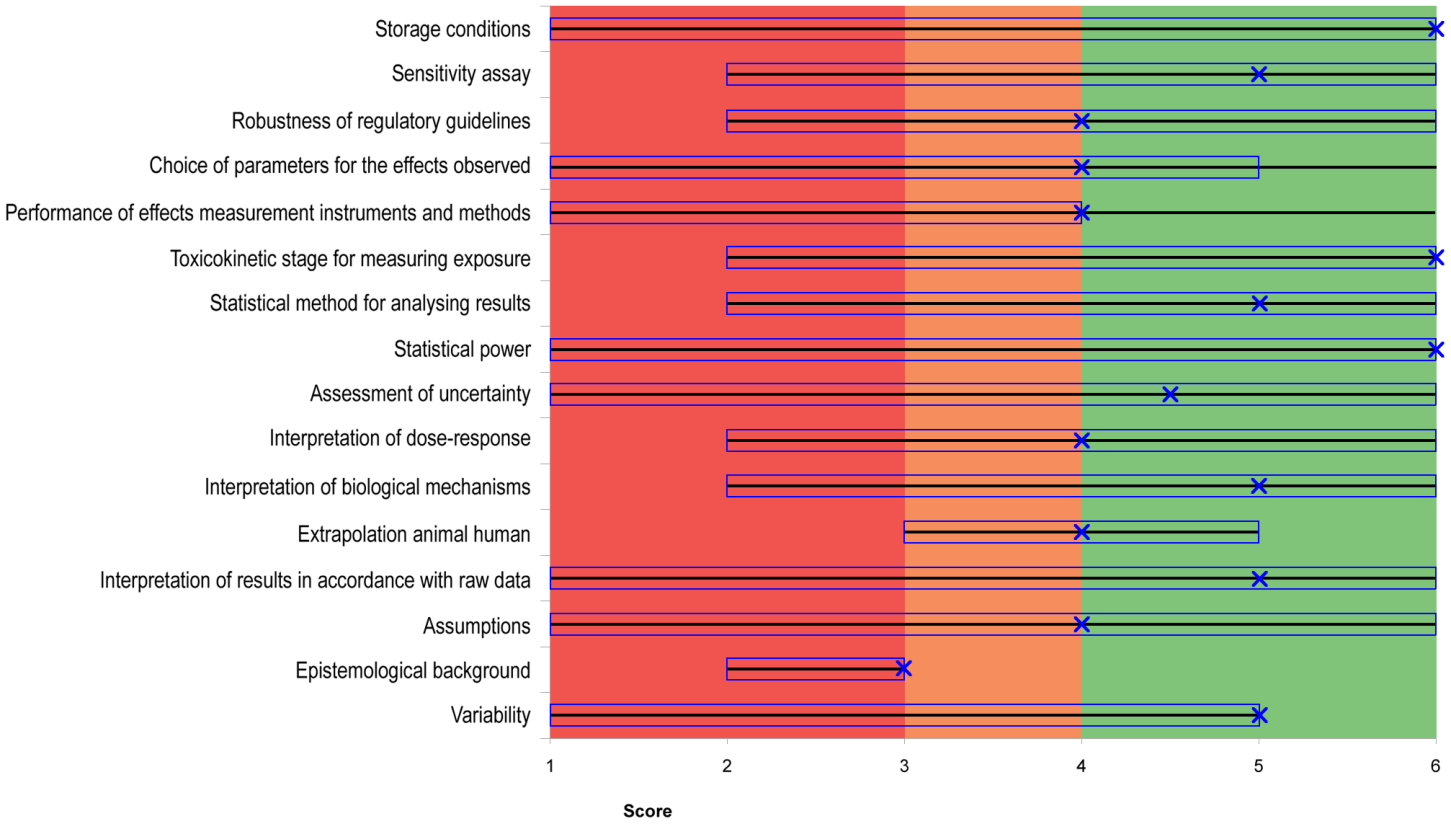
Quality assessment of Stump (2009), using Qualichem with four respondents. For the report of Stump (2009), of the possible 45, the figure represents only the 16 controversial criteria. All the other criteria—those that are not controversial according to our definition—received scores of 5 or 6 and were considered as having a high aggregated quality. The figure is divided in three colored areas: red (including scores and medians <3), orange (for scores and medians between 3 and 4) and green (for scores or medians >4). A line covers the full range from the lowest score to the highest score in the group of responding experts. The median of the scores is represented by an “x” and the interquartile range is represented with a rectangle. If the median (x) is in the red area, the aggregated quality of the criterion is low. If the median is in the orange area, the aggregated quality is average. If the median is in the green area, the aggregated quality is high. The interquartile range is an indicator of inter-expert heterogeneity. Nine of the 16 controversial criteria were of high aggregated quality (median fell in the green area). The remaining 7 criteria were all of average aggregated quality (median fell in the orange area).

**Figure 3:**
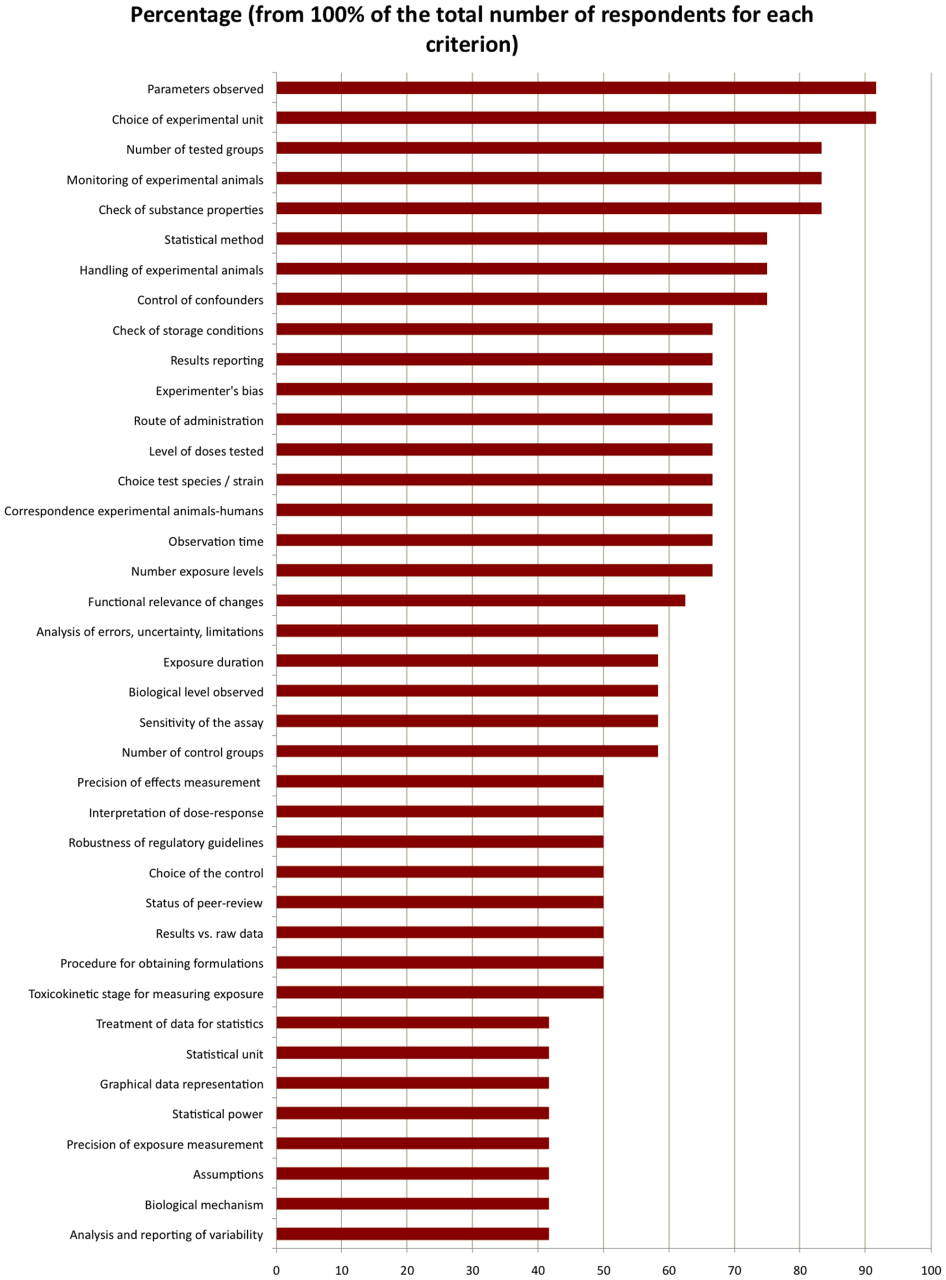
Relative importance of the Qualichem quality criteria to the global quality of the study. Eight of the twelve respondents agreed to select a subset of up to 15 criteria that they considered to be the most important for the quality of *in vivo* study results. The figure shows the combined 39 criteria chosen by these eight experts. The vertical axis represents the 39 criteria, and the horizontal axis represents the percentage of respondents that selected each criterion.

In other words, if the “x” in figures 1, 2 and 3, is in the red area, the aggregated quality of the criterion is low. If the “x” is in the orange area, the aggregated quality is average, and if it is in the green area the aggregated quality is high.

The interquartile range shown with a rectangle on the graphical representation is another indicator of inter-expert heterogeneity.

**Critical criteria** are a subset of controversial criteria, and are used to calculate a multi-expert aggregated level of confidence in the study. The term “level of confidence” has a very precise meaning in statistics; however, in this paper we use “level of confidence” to referring to the quality of *in vivo* studies. We used this wording because our experience is that this formulation is easy to understand and is common wording for experts in health agencies [15].

### 2.6. Definition of critical criteria: a measure of the level of confidence in a study

A higher quality study will have high scores on more criteria. Depending on the scores given by the respondents, some criteria might play a greater role than others in determining the overall quality of the study. **Critical criteria** are defined as those controversial criteria of which the scores meet at least one of the following conditions:

- they are very heterogeneous—there is a difference of 4 or 5 points between any two respondents (the maximum possible difference between the scores of two respondents is 5);
- they are very low scores—at least one respondent gave a score of 1; or
- they show low or average aggregated quality—the median of scores is ≤4 (the “x” in the red or orange area, Fig. 1 and 2).

To define an **overall level of confidence in a study**, we established a decision rule based on the number of critical criteria. A study has:

- **a high level of confidence** if less than one-third of criteria (≤14/45) are critical
- **an average level of confidence** if between one-third and two-thirds of criteria (15 to 30/45) are critical
- **a low level of confidence** if more than two-thirds of criteria (≥31/45) are critical

This decision rule is based in the assumption that all criteria have an equal weight, which may not be valid (see discussion). Additional decision rules could be established, and testing these decision rules could be the object of further research, before the method is standardized.

### 2.7. Relative weights of different Qualichem criteria

We assessed the relative weight of each Qualichem criteria in determining the overall (aggregated) quality of the studies. Our respondents were given two options: a) indicate that all criteria are equally important, and b) choose a maximum of 15 criteria that are the most important for the overall quality of the results.

### 2.8. Influence of experts' affiliation and background/expertise on the use of Qualichem

Each of the respondents was asked to fill in an “expert profile” (Text S5) designed to identify their discipline, their publication activity (particularly on BPA and endocrine disrupters), the nature of their knowledge of BPA (i.e., experimental and/or theoretical), whether their expertise is specialized on BPA and/or endocrine disrupters or generalized on toxicology or other areas, and their institutional affiliation and financial links with industry.

We did not expect a statistical correlation between respondents' characteristics and their responses. However, these characteristics could influence their expert judgments about study quality. For the purpose of comparison, we isolated and graphically represented affiliation or disciplinary clusters in a separate figure. We created this representation for the respondents who included “endocrinology” or “endocrine toxicology” among their fields of competence. Also, we compared the results of this cluster, i.e., criteria in the red/orange area, with the results obtained on all the respondents together. Significant, easy-to-observe differences can be interpreted as an indication of the influence of discipline/affiliation/interests.

For the future use of Qualichem, any other disciplinary, conflicting interests or affiliation clusters can be similarly isolated and compared. Such a separation can be done easily using the internet-based version of Qualichem available at URL: http://www.qualichem.cnrs.fr/.

## Results

### 3.1. Two *in vivo* case studies that assess the effects of BPA

This section presents the Qualichem results for the two case studies: Tyl et al. (2002) and Stump (2009).

As a reminder, the graphical representations of Qualichem (Fig. 1 and 2) include only controversial criteria. The assessment of the level of confidence in a study using Qualichem is based on the subset of those controversial criteria that are considered critical.

The average length of an interview was two hours—about 90 minutes of that was required to fill out the Qualichem survey. We assume an additional two to four hours was required for the respondent to read the study before the meeting. However, in some cases, the time for analyzing some particular studies might be much longer, e.g., when re-analysis of original raw data is done. However, we estimate that this applies to particular situations and is not the regular case of peer-reviews.

#### 3.1.1. Qualichem in vivo for Tyl et al., 2002

Figure 1 represents the application of Qualichem to the Tyl et al. (2002) study—eight respondents participated (Text S8). The respondents provided justification for why they assigned a score to each criterion, and these are presented in Text S3. Our goal was to synthesize their explanations without critically commenting on them. For example, bibliographic references were not included unless the respondents themselves provided them.

Of the possible 45, 35 controversial criteria were identified using Qualichem. In most cases in which a six was assigned to a criterion, it was either because the study respected regulatory guidelines or because a respondent had personal experience with relevant current practice.

The median score for a given criterion, as a reminder, represents its aggregated quality. Thirty of the 35 controversial criteria were of high aggregated quality (the median is in the green area). Of the five remaining criteria, three were of average aggregated quality (the median is between 3 and 4 and in the orange area) and two were of low aggregated quality (the median is in the red area).

Twenty-seven of the controversial criteria were also identified as critical, which is more than one-third of the 45 criteria. As such, confidence in Tyl et al. (2002) can be considered average. This contradicts official evaluations of this study by the Scientific Committee on Food [30] and the European Chemicals Bureau (ECB) [31], who considered it a very good quality, “pivotal” study, and suitable to use to determine the NOAEL of BPA.

#### 3.1.2. Qualichem in vivo for Stump (2009)

Figure 2 represents the application of Qualichem to the report by Stump (2009) (Text S9). Despite the large number of competent scientists contacted by email (64), only four respondents agreed to participate. Because the objective was to test the tool, it is not the number of experts for each case study that is important, but the total number of experts that participated in testing Qualichem. The total number was twelve, which is well within the typical goal range in expert elicitation studies (6 to 12 experts [27]).

The respondents provided justification for why they assigned a score to each criterion, and these are presented uncritically in Text S3.

Of the possible 45, 16 controversial criteria were identified using Qualichem. Nine of these controversial criteria were of high aggregated quality (median fell in the green area). The remaining seven were all of average aggregated quality (median fell in the orange area).

All 16 of the controversial quality criteria were also identified as critical. Because between one-third and two-thirds of the criteria were critical, the study can be labeled as providing an average level of confidence, according to our decision rule. This characterization contradicts the evaluation made by the EFSA [32], who rejected the study.

#### 3.1.3. Two levels of quality

We distinguished two levels of quality, i.e., aggregated quality and level of confidence for the whole study. They provide a way to represent both majority and minority opinions, and they should be read together. For both studies, most criteria show high aggregated quality (median in the green area), which indicates that most scores were favorable. However, both studies also receive only an average level of confidence, which shows that minority opinions are numerous and important.

In the next section, we compare the criteria used by SCF, ECB or EFSA with those of the Qualichem typology to explore the reasons behind their contradictory evaluations for the two studies.

### 3.2. Assessment of study quality in safety agencies

Though these two studies may have been assessed by national health agencies, we focused on comparing Qualichem with quality assessments done by European institutions. These institutions play an essential role in advising decision-making on regulatory values for exposure to BPA in European countries, and it is relatively easy to access their documents.

#### 3.2.1. Tyl et al. (2002)

The Tyl study was used in the Opinion of the Scientific Committee on Food [30] as a pivotal study for deriving an NOAEL for BPA. This study was thought to be of good quality because of its long observation time, use of a high number of doses, and inclusion of parameters specific to endocrine disruptors such as anogenital distance, acquisition of puberty, estrous cyclicity, sperm parameters and nipple retention in males. The effects on body weight and some organ weights seen by the authors at 50 mg/kg bw/day were considered to be relevant; therefore, the NOAEL was set at 5 mg/kg bw/day. No quality weakness was highlighted in the SCF assessment of the study.

Just one year later, the ECB reinterpreted the raw data, leading to a change in the NOAEL. In opposition to the SCF [30], the ECB excluded the effects found at 50 mg/kg bw/day by considering them not “*consistent*” [31] (p. 180). The ECB concluded that “*overall, this study showed 500 mg/kg bisphenol-A causes a reduction in the number of pups per litter*” and that “*the NOAEL for both parental and reproductive toxicity is 50 mg/kg/day*”. The ECB considered the study of high quality, and referred to it as “*well conducted and reported*” (pp. 179), “*comprehensive, good-quality multigeneration*” investigation, and also referred to the use of an OECD guideline (p. 214). An illustrative detail is that this reference to the OECD two-generation reproduction toxicity study guideline (probably OECD 416) is incorrect, because the study actually followed an OPPTS guideline [34], and the two guidelines are not identical.

The 2006 EFSA report confirmed the NOAEL of 5 mg/kg bw/day, which was considered adequate and in accordance with a more recent, modified OECD 416-guideline study on mice [35]. The EFSA Panel considered that this NOAEL, “*identified in the SCF evaluation of 2002 is still valid*” [36] (p. 6).

#### 3.2.2. Stump (2009)

In contrast to the Tyl study, for which detailed analysis of the strengths and weaknesses of the study is not available in regulatory documents, EFSA dedicated a whole report to the Stump study. Below, we compare quality criteria addressed by these documents with the results produced with Qualichem.

For six of the 16 critical quality criteria identified with Qualichem, similar assessments were done in_the EFSA reports [32]–[33]:

- Regarding the **sensitivity of the assay**, the Biel maze was characterized as not having the “*potential to demonstrate equivalence of BPA compared to a control*”, because EFSA's Assessment and Methodology Unit revealed “*an extreme high variability, not only in the study data for the PND62 trials, but also in the data for PND22. This variability might be due to other non-modelled or not-possible-to model aspects of the experimental design or execution of the experiment*”. However, in contrast to Qualichem, this criterion was given very high weight by the EFSA working group—decisive over all the others—and was considered sufficient for declaring: “*therefore, this study should be considered as inconclusive*” [32] (pp. 25).
- The **choice of the parameters observed** was also found to be incomplete by the EFSA working group (WG), whose experts identified missing aspects of behavior related to anxiety, avoidance learning, schedule-controlled behavior, sexual dimorphic behavior and impulsiveness. Nevertheless, the WG considered this criterion less important than the sensitivity of the assay, as it was considered not sufficient to invalidate the study. Qualichem scores for this criterion ranged from 1 to 6, with the median in the orange area (4).
- The **interpretation of results as compared to raw data** was comparable between Qualichem, (scores from 1 to 6) and EFSA. Those aspects where a difference between the raw data and the authors' interpretation was identified—namely, effects of BPA on gross motor movements; convulsions and seizures; and censored and variable data on learning and memory from the Biel maze [32]–[33] were declared inconclusive and the respective parts of the study were considered unusable.
- The **epistemological background** was found to be lacking mechanistic knowledge, in particular on the interaction between BPA and estrogen receptors [32]. Qualichem produced a similar result, with the median score in the orange area (3).
- The treatment of **assumptions** was both a critical quality criterion in Qualichem (Fig. 2) and repeatedly addressed by EFSA. Qualichem respondents were not very precise, but expressed that assumptions were not reported adequately. On the contrary, EFSA [33] was very specific (pp. 7), stating that the analysis was “*based on very general hypotheses*” about the categorical variables of the model, the effects were potentially random, and the shape of the distribution of the response variable may not have been normal.
- One of the most important critiques brought by the EFSA [32]–[33] reports was about **variability**. In the learning and memory results, the variability was considered too high for any conclusion to be drawn. In contrast, the expected variability in motor activity between males and females was not found. Variability also seems to have been considered very important for rejecting the whole study. One of the Qualichem respondents gave a minimal score of 1 for this criterion, while the other three assigned scores of 5 or 6.

As well, the value of seven other quality criteria assigned by EFSA [32] opposed our Qualichem results:

- For the **choice of the control**, our four respondents felt that a negative control was justified, and assigned scores of 5 (1 respondent) and 6 (three respondents). In contrast, EFSA [32] considered that “*an oestrogenic reference compound was not included. Therefore, sensitivity of the test parameters to oestrogenic substances has not been demonstrated*” (pp. 19). Similarly, EFSA (2010b) considers that “*as no positive control group was inserted in the Stump et al. (2010) study it is not possible to know if a positive effect could have been statistically picked up if one truly existed”* (pp. 6). However, this continues with another statement, more in line with Qualichem responses: “*however, it should be noted that no generally accepted reference compounds are available for this purpose*” (pp. 22). The Stump (2009) study was published in 2010 in the journal Toxicological Sciences [37].
- **Results reporting** was often criticized by EFSA [32]–[33], referencing incomplete data for surface righting response, ambulatory count, or random effects for litter on PND62. Furthermore, some aspects of the protocol are not clear; for example, the definition of “errors” in the Biel maze [33]. Two Qualichem respondents gave maximal scores of 6, saying they felt the data reporting was exhaustive. The other two gave scores of 5, noting the statistical methods used were complex and difficult to understand. These high scores indicate that reporting was not considered to be a critical quality criterion by the Qualichem respondents. The EFSA WG did not quantify this criterion so it is difficult to compare the importance given to it.
- **The choice and treatment of data for statistical analysis** and **the choice of the statistical method** were heavily criticized by the EFSA experts because of the lack of statistical treatment of the time-to-escape data, the data for long-term memory effects, and of data censoring. EFSA [33] considered that “*the Biel maze had not been appropriately statistically analyzed and therefore conclusions drawn from the results of these analyses cannot be relied upon*” (pp. 4). Furthermore, pooling the results diluted the possibility of finding effects at some specific moments in time. With regard to data treatment, two of the Qualichem respondents declined to answer due to a lack of specific statistical competence. The two others gave maximal scores. Three Qualichem respondents answered the question about the choice of statistical analysis. One assigned a 2, giving arguments similar to EFSA's. The two other were uncritical, giving scores of 5 and 6.
- The EFSA WG considered the **graphical representation of the data** inadequate for some results from the Biel maze test, saying it impeded interpretation and comparison of the slopes. In contrast, all four Qualichem respondents gave maximal scores for this question.
- EFSA also considered **the choice of the observation time** insufficient (3 minutes for the Biel maze) [32]–[33]. All four Qualichem respondents gave maximal scores, referencing the total time of observation in the experiment (three generation, but ignoring the time of observation for particular parameters).
- **Coherence with other studies** was considered absent for popcorn seizures and therefore a sufficient reason to cast “*doubt on the relevance of this observation*” [32] (p. 28). Both the study by Stump and the rest of the existing literature on neurobehavioral toxicity were considered too low quality to be used to assess the effects of BPA. Three of the Qualichem respondents gave maximal scores, indicating that they considered the results of the study in line with other robust published results. Two respondents did not provide justification for their scores, but a third said that the Stump study was in line with other robust studies, even if it contradicted other studies considered to be of low quality. The fourth respondent could not answer, arguing that no other comparable study, in terms of number of animals and doses employed, was available in the literature.
- **Experimenter's bias** was potentially associated with the imprecise definition of “error” in the Biel maze test. According to EFSA [33], “*this definition is imprecise as it could be subject to different interpretations*” (pp. 6). None of the Qualichem respondents noted this.

A criterion of “reproducibility” was used by EFSA [36] to argue for rejecting studies that indicate low dose effects of BPA: “the Panel considered that low-dose effects of BPA in rodents have not been demonstrated in a robust and reproducible way, such that they could be used as pivotal studies for risk assessment” (p. 4). This criterion has been defined rather vaguely: “low-dose effects on specific biological endpoints have been reported in some studies, but were not replicated in others” (p. 43). We have tested the use of “reproducibility” for assessing the quality of a study including a specific question in our Qualichem protocol. However, it was very difficult or impossible for our respondents to answer that question. They considered that studies could not be identically reproduced in toxicology, because the particular conditions of a specific experiment cannot be identically reproduced in another, even if an explicit aim is to confirm the results. Furthermore, toxicologists have no incentives to repeat previous studies, given that publication criteria and research funding are based on originality. The meaning given by EFSA [36] to reproducibility and the rationale for giving this criterion significant weight in its assessment of the low dose literature therefore remains unclear.

There were no quality criteria discussed in any of the SCF, ECB or EFSA reports above that was not dealt with in our typology.

### 3.3. Are Qualichem criteria already addressed in existing guidelines and in REACH?

Tyl et al. (2002) meets the OPPTS 870.3800 standardized guideline on reproductive toxicity [34], corresponding to OECD 416 [38]. Stump (2009) claims to comply with the guidelines OECD 426 [39] and OPPTS 870.6300 [40] on developmental neurotoxicity. For certain Qualichem quality criteria, these standardized guidelines indicate best practices. For others, the guidelines vary. They may be flexible and leave choice of method to the discretion of the experimenters, address methodology briefly without giving precise indications, ask experimenters to report “what” they do but not prescribe “how” it should be done, or not address methodology at all.

In Text S6, we identify the Qualichem quality criteria that are addressed (with varying levels of precision) in the four standardized guidelines relevant for the two studies. In addition, we compared Qualichem criteria with information that REACH demands of industry and with the classes the ARRIVE guideline recommend for the scientific communication of *in vivo* studies [9]. This comparison also aimed to check the assumption, made by some of our respondents, that respecting regulatory guidelines ensures scientific quality for an *in vivo* study. As Text S6 shows (summarized in table 3), this assumption is not realistic—only some quality criteria are addressed in a precise way in standardized guidelines, with clear experimentation procedures to follow. Other quality criteria either depend on the choices made by experimenters or are not addressed at all. Finally, GLP [41]–[42] only allows traceability of laboratory procedures and limits the possibility of fraud in private laboratories [13], but it is not a standard of scientific quality.

**Table 3:** Comparison between Qualichem and other reporting and/or quality assessment frameworks.

Reporting and/or quality assessment framework	REACH registration, on-line version	OECD 416 guideline	OECD 426 guideline	OPPTS 870.3800 guideline	OPPTS 870.6300 guideline	GLP	Expert committees in safety agencies (SCF, ECB, EFSA)	ARRIVE guideline
Number of criteria addressed in each framework, out of the 45 Qualichem criteria	22/45	20/45	25/45	16/45	17/45	18/45	21/45	21/45

There are no quality criteria in documents produced by expert groups or in the OECD 416, OECD 426, OPPTS 870.3800, OPPTS 870.6300 or GLP guidelines that are not in Qualichem (Text S6). This is related to the method used for developing Qualichem criteria—it began with criteria already present in safety agency reports, and completed them based on analysis of public criticism made by other stakeholders and on feedback from our respondents.

### 3.4. Relative importance of the different criteria for the global quality of the study

We assessed the relative importance of the different Qualichem criteria in determining the final quality of the studies. Four respondents chose option a) indicate that all criteria are equally important, and eight chose option b) select a maximum of 15 criteria that are the most important for the final quality of the results.

Figure 3 shows the 39 criteria chosen by at least one of the eight respondents who chose option b, in percentage of respondents that considered each criterion important.

75% or more of the respondents considered 8 of the 45 quality criteria as high priority, 60% or more of the respondents considered 18 criteria as high priority and 50% or more of the respondents considered 31 criteria as high priority.

### 3.5. Influence of respondents' disciplinary background and publication history on their quality assessments

**Figure 4:**
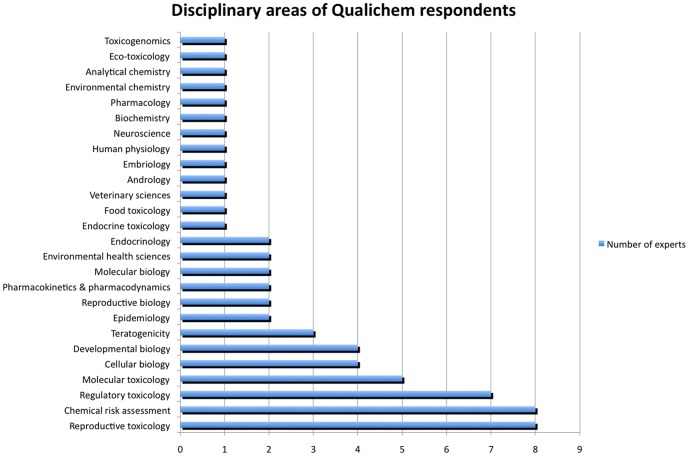
Disciplines of Qualichem respondents. The vertical axis represents the disciplinary areas chosen by the experts. The horizontal axis represents the number of experts who chose each disciplinary area to describe his/her work.

Four respondents were employed by safety agencies and the other eight were academics. Nine were, at some time, part of official expert committees involved in the assessment of BPA at different levels and in different countries in Europe. We did not have information about participation in expert committees for two respondents. Among the 12 respondents, three had not published on BPA in a peer-reviewed journal since 2006. We were not able to access a list of publications for another three (one academic and two employees of safety agencies). Six others have published at least one article addressing BPA at different levels of detail, in a peer-reviewed journal, since 2006. Figure 4 shows the disciplinary areas of the respondents—they were allowed to select multiple options.

Interestingly, three of the four respondents who work in safety agencies gave scores under 5 for the criterion “Robustness of regulatory guidelines”. The fourth was the only respondent who gave maximal scores to all quality criteria for the study assessed. This person reiterated that following a guideline is in itself a guarantee of quality, and noted that he knew the work of the people who conducted the study and trusted them. One academic scientist made a similar statement, saying that following a guideline is a sufficient guarantee of scientific quality—this person gave only one score of less than 5, for “general state of scientific knowledge”. The number of quality criteria assigned scores under 5 was similar for the safety agency and academic respondents, indicating similar levels of criticism.

**Figure 5:**
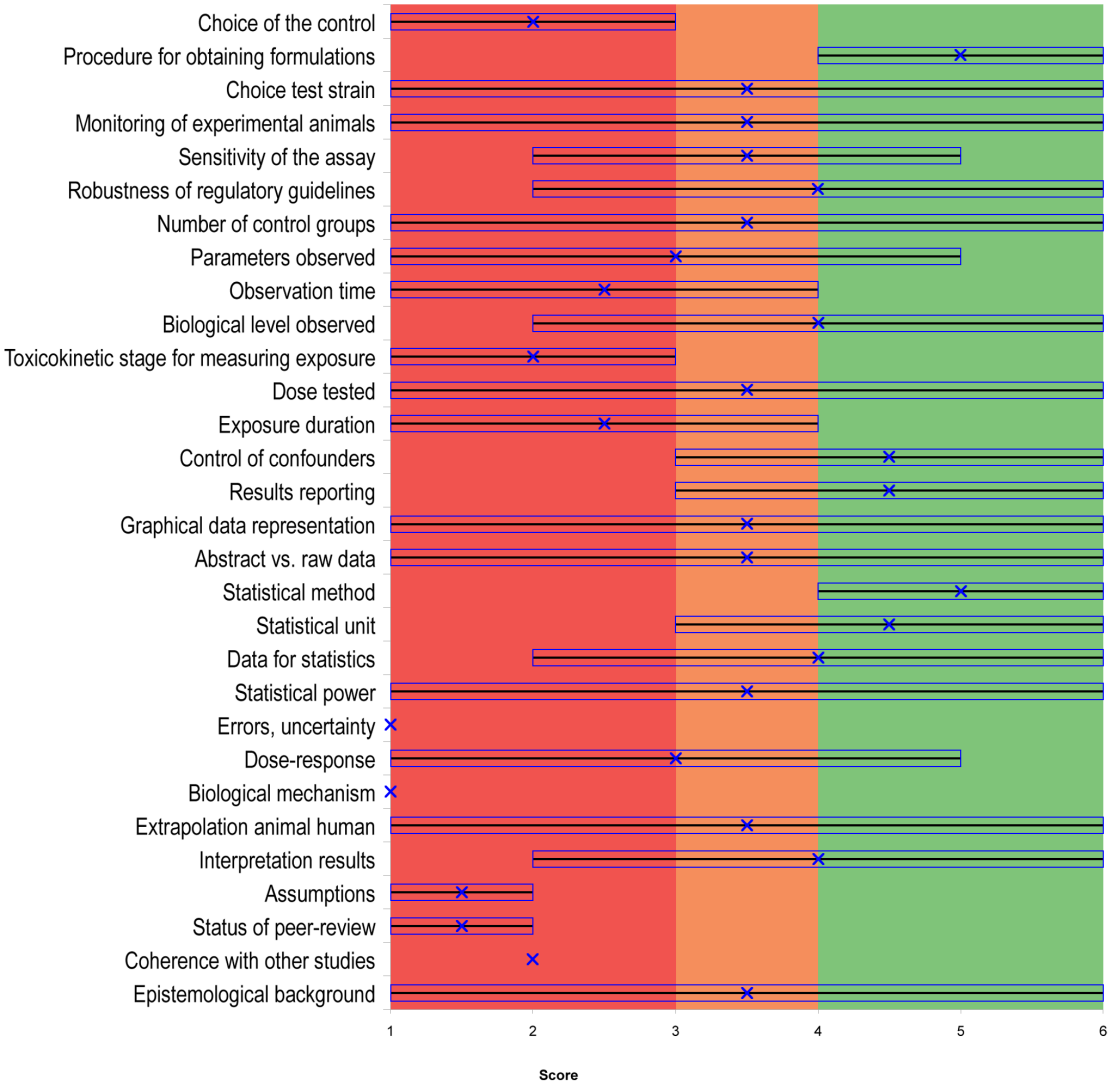
Quality assessment by two endocrinologists using Qualichem to evaluate Tyl et al. (2002). For the study of Tyl et al. (2002), of the 45 criteria, the figure represents only the 30 controversial criteria for the two respondents who included “endocrinology” or “endocrine toxicology” among their fields of competence. The figure is divided into three colored areas: red (including scores and medians <3), orange (for scores and medians between 3 and 4) and green (for scores and medians >4). A line covers the full range from the lowest score to the highest score in the group of responding experts. The median of the scores is represented by an “x” and the interquartile range is represented by a rectangle. If the median (x) is in the red area, the aggregated quality of the criterion is low. If the median is in the orange area, the aggregated quality is average. If the median is in the green area, the aggregated quality is high. The interquartile range is an indicator of inter-expert heterogeneity. The number of criteria that fell in the orange or red areas is much higher for these two respondents than for all respondents together: 16 vs 3 in the orange area, 9 vs 2 in the red area, and only 5 vs 30 in the green area. This indicates lower levels of aggregated quality for these criteria, compared to the 8 respondents together.

The three most critical respondents gave scores lower than 5 to 14, 15 and 21 quality criteria. These were also the only respondents who included “endocrinology” or “endocrine toxicology” among their fields of competence. Representation of Qualichem (Figure 5, Text S10) for only two of these academic scientists (they assessed Tyl et al. [2002]) shows that the number of controversial criteria (30 vs 35) and critical criteria (25 vs 26) for these two respondents combined is similar to those given by the eight respondents together. However, the number of quality criteria that fall in the orange or red areas is much higher for these two respondents than for all respondents together: 16 vs 3 in the orange area, 9 vs 2 in the red area, and only 5 vs 30 in the green area. This indicates lower levels of aggregated quality for these criteria, compared to the eight respondents together.

## Discussion

### 4.1. Heterogeneity in experts' quality assessments and working procedures in safety agencies

Our results show substantial heterogeneity among scientists in how they evaluate the quality of a study using the same criteria, and also differences between Qualichem assessments and those done by previous expert committees. This heterogeneity is not accounted for in an appropriate manner in current working procedures used by expert committees in health agencies.

A number of factors could explain these differences. Experts may not have time to familiarize themselves with the important details of a study, may view quality differently or may just be wrong. Pragmatic considerations related to lack of time or resources can also influence how thoroughly a study's quality is evaluated.

In expert groups, consensus-based procedures sometimes favor strong personalities who take the lead in collective discussions—important minority opinions can be lost in the process of reaching a common position. Individuals can also be reluctant to express critical opinions in a group context, in particular when those opinions disagree with the group majority and/or president—a phenomenon called “the spiral of silence” [43]. When individual views are not heard or expressed, the overall quality of an expert group's work can be compromised. A minority opinion in an expert committee is not necessarily a minority opinion in science, but could result from the criteria used for selecting experts, which could favour certain disciplines and competences over others. Furthermore, communicating the result of a quality assessment as a consensus masks certain quality problems, and can cause decision-makers to view a study's results as being stronger than they really are.

The institutional context of certain safety agencies, despite recent efforts, favors consensus and remains resistant to minority opinions. We know of two examples within our personal contacts of situations at EFSA in which one expert wanted to express a minority opinion but was discouraged from doing so. Qualichem avoids these problems by providing two indicators of quality: a majority-based indicator for each criterion (aggregated quality) and a multi-expert, aggregated level of confidence in a study. This allows each individual in an expert group to communicate his/her critical position, even if this position is minority.

### 4.2. Lowering subjectivity in regulatory quality assessments

The current regulatory framework in Europe (e.g., REACH and the Regulation (EC) no 1107/2009 concerning placement of plant protection products on the market) demands that industry assess the risks of the chemicals it produces. Therefore, industry studies are frequently considered by health agencies. Research has repeatedly confirmed that conflicts of interest can bias results in favor of a study's sponsor [44], [1]. The ability of health agencies to evaluate work done by industry would benefit from rigorous quality assessment, with comparable criteria to evaluate both academic and industry studies and clear evaluation methods—an improvement over relying on fuzzy “expert judgment”. Of course, providing a score from a scale without explanatory arguments can be as subjective as any other unstructured “expert judgment”. For this reason, the Qualichem protocol requires respondents to support their choice of score for each quality criterion with one or more arguments that are documented together with the score (Text S7).

Furthermore, Qualichem could be further improved by adding a requirement that respondents provide scientific references from the peer-reviewed literature, where available, to support their choice of scores. There are currently unstandardized practices in health agencies on referencing expert arguments [45]. However, expert judgment can be erroneous or biased if arguments are not referenced by peer-reviewed literature—a phenomenon that tends to be important in socio-politically controversial cases [46].

The advantage of Qualichem is that it represents each quality criterion, which cannot be understood solely from a narrative description such as is presented in the SCF, ECB and EFSA reports. Furthermore, Qualichem provides a common framework that applies the same quality criteria for each study assessed and allows for quick comparison between studies and between health agencies, as shown here for our two cases. Indeed, without a common background for assessing the quality of available knowledge, different expert committees can reach opposite conclusions (e.g., “risk to all or none”) based on the same data [47]. Such situations fuel controversies, create doubt and suspicion, reduce the legitimacy of official risk assessments, give an impression of lack of transparency, waste public money by necessitating multiple expert groups, and delay decision-making.

In the framework of REACH, a structured assessment of the quality of the studies submitted by industry could reduce the potential influence of conflicts of interest and provide a systematic approach that could facilitate the work of health agencies. It is currently difficult to access the original industry studies. Indeed, safety agencies do not have systematic access to the complete reports of the all studies communicated by industry in their registration dossiers. To access these full reports, safety agencies must sometimes negotiate with industry and ask for them. But, there is no guarantee that industry will provide the full reports. Access to raw data for re-analysis is even more problematic. Furthermore, some study reports can be quite thick, and raw data and results are not always reported in an understandable manner, which can significantly delay the process of evaluating a study. A summary of the most relevant information contained in a report is essential to efficiently shorten the time for evaluating its quality.

In the on-line version of REACH registration, industry can submit their studies according to a pre-established framework that includes administrative data, data source, materials and methods (i.e., test type, test guideline, GLP compliance, test materials, test animals), administration/exposure, examinations, results and discussions, further observations and conclusions. As shown in Table 3 and Text S6, the information currently provided in the on-line version of REACH registration dossiers for the two studies assessed includes only half of the Qualichem criteria. If information on all the Qualichem criteria were available online, an industry study could be more easily reviewed.

Qualichem for *in vivo* studies is based a generic procedure for developing quality assessment instruments and can be generalized to other types of regulatory productions. Currently, we are testing similar tools for epidemiologic studies, for risk assessment reports produced by health agencies, and for exposure characterization.

Finally, Qualichem provides a basis for precise, reproducible and transparent assessment that could replace the Klimisch scores, which are currently subject to significant subjectivity and a confusing valuation procedure [12].

### 4.3. Quality assessment and standardized guidelines

Currently, regulatory guidelines and the GLP standard are given important weight in assessing the quality of studies in regulatory toxicology. While two of twelve respondents of our study stated from the very beginning of the interviews that respect of regulatory guidelines is an undisputable guarantee of scientific quality, in-depth evaluation led to very different quality assessments for the others. A study that respects regulatory guidelines can still present quality failures that can be judged important by certain scientists (Figures 1 and 2). Furthermore, regulatory guidance does not address several criteria that scientists consider important for assessing the quality of studies (Table 3, Text S6). For other criteria, required standards are designed to ensure the quality of work at a gross level; however, they do not account for the relevant particularities of specific substances, such as parameters for measuring neurotoxicity. Most of the criteria that are not well addressed in regulatory guidelines were considered by our Qualichem respondents as having an important weight for the final quality of the study (Figure 3 and Text S6): control of confounders; correspondence between the experimental animals and humans; interpretation of the functional relevance of behavioral, morphologic, histological, molecular or biochemical changes; analysis of errors, uncertainty and limitations; sensitivity of the assay; status of peer-review; interpretation of raw data; choice of the toxicokinetic level for measuring exposure; data treatment for statistics; graphical representation; statistical power; precision of exposure measurement; analysis of assumptions; analysis and reporting of variability.

Indeed, regulatory guidelines change very slowly. For example, development of the OECD 426 guideline took 12 years [48]. Their objective is not to reflect the best scientific knowledge, but to offer a science-based political compromise among OECD member states. For this reason, there is significant potential for a gap between some OECD guidelines and rapidly advancing scientific knowledge. This is currently one of the major reasons for sociopolitical controversy about endocrine disrupters. In addition, the guidelines allow flexibility about certain aspects of the experimental protocol and leave the experimenter open to interpret the current state of scientific knowledge. However, this flexibility also allows room for experimenters to be wrong or to choose a level of scientific robustness that does not reflect available knowledge.

Furthermore, most academic studies that could be useful for decision-making do not follow OECD or GLP guidelines and therefore start off significantly disadvantaged when it comes to regulatory assessment of the study's quality. Robust science-based decision-making requires a more balanced playing field that considers both industry and academic studies. Systematic evaluation of studies as they relate to the current state of scientific knowledge is needed for well-informed decision-making, and to overcome the inevitable time delays in adapting OECD guidelines. Safety agencies do this kind of evaluation, but not in a systematic, transparent and comparable way from one agency to another.

Guidelines have a good regulatory reputation for providing scientific quality [13]. For example, the Klimisch score calculates four levels of reliability for a study: reliable without restrictions, reliable with restrictions, not reliable, not assignable. The highest score for reliability (reliable without restrictions) is received by studies that were carried out according to standardized testing guidelines.

However, standardized guidelines only partially deserve this reputation (see Table 3, Text S6 and respondents' criticism on the “robustness of regulatory guidelines” in Fig. 1 and 2). This finding is in line with previous literature that suggests that some endpoints of the OECD 426 guideline, such as assessment of cognitive and sensory dysfunction, are not adequately sensitive while others are overly sensitive. In addition, specific endpoints like social behavior, pharmacokinetics and neurochemistry are lacking, and there is significant variability in the endpoints that are defined, like motor activity [48]. The use of standardized guidelines as a major indication of scientific quality is currently controversial [48] [13].

For all these reasons, the use of standardized guidelines should not replace scientific quality assessment, but should be considered in a complementary way, as we have suggested in our interview protocols.

### 4.4. Relative weights of criteria in quality assessment

The weight of the same criterion could differ between studies and contexts, e.g., academic publication or regulatory assessment. An approach that asks respondents to weight each criterion, in addition to their Likert score, could be developed and tested in further work.

Also, the calculation of the level of confidence could depend on the number of respondents involved, as our algorithm to identify critical criteria gives important weight to each individual respondent.

There are several reasons for the contradictions between our Qualichem assessment and the evaluations made by SCF, ECB and EFSA. We showed that quality criteria used by official expert bodies differ from, and represent only a subset of, those included in Qualichem. Also, previous expert groups analyzed in this paper gave different weights to certain criteria than did Qualichem respondents.

In addition, group dynamics and procedures in official consensus-based expert groups leave only limited space for individual experts to express their insights on uncertainties and for these to be incorporated in the group's final conclusion. Qualichem keeps track of each respondent's criticism and values individual assessments by using the decision rule that defines critical criteria and, in turn, the ultimate level of confidence in a study.

The expert groups that previously evaluated the two studies were inevitably different from Qualichem respondents, in terms of range of disciplinary domains and experience with endocrine disrupters and BPA. Some respondents are likely able to react more to some quality criteria and less to others. Our interviews showed that some respondents were not able to assess all quality criteria and responded “cannot answer”. This assumption is further reinforced by our results (section 3.5) showing significant differences between endocrinologists and other respondents, which reiterates previous results that indicate different approaches to endocrine disrupters in toxicology versus endocrinology [49].

Evaluation of each of the Qualichem criteria depends on the state of knowledge at the moment of the evaluation. Applying Qualichem at the same time as the expert groups did their reports (2002, 2003 or 2010) would probably have given different results. Therefore, the most important result from our study is the Qualichem method itself; the two case studies have been used primarily to test and demonstrate the method.

Our work is in line with the proposals of Evidence-Based Toxicology [50]. Using Qualichem, the level of confidence in a study could be established on a clear and comparable basis, by including the views of all experts involved, including minority views. Respondents could express themselves naturally without needing cumbersome procedures such as “minority opinions”. Reporting both consensus and controversial points could facilitate discussion in expert groups, and allow for easier representation of the quality of studies for health agency employees.

## Conclusions

There is currently no clear and reproducible procedure for evaluating the quality of studies in regulatory expertise. Though considering a study of chemical risks valid or rejected can have tremendous consequences on the lives of people exposed to those risks, quality assessment remains unstructured, cannot be compared among expert groups and agencies, and cannot be transparently communicated. Respect of standardized OECD and GLP guidelines is currently considered a token of scientific reliability, but this is becoming more controversial.

We have developed a tool, called Qualichem *in vivo*, to systematically and transparently assess the quality of *in vivo* studies used in chemical health risk assessment. Qualichem was usable for both short (a few pages) and extensive (4,000 page) study reports. For both studies, Qualichem contradicted the quality assessments done by expert committees in safety agencies, and confirmed that standardized guidelines only partly deserve their reputation as providers of scientific quality.

Our study shows four main results:

- the 12 respondents considered the Qualichem criteria as appropriate for quality assessment of *in vivo* studies
- there is important heterogeneity among experts in their quality assessments, which is not well accounted for in current working procedures in health agencies
- standardized guidelines do not appropriately include important quality criteria
- different criteria have different weights for the final quality of a study.

Qualichem provides two indicators of quality: a majority-based indicator for each criterion (i.e., aggregated quality) and a multi-expert, aggregated level of confidence in a study that allows each individual in an expert group to communicate his/her critical position, even if this position is a minority one (i.e., the global level of confidence in a study).

Our results indicate different levels of confidence from official quality assessments of two studies. Tyl et al. (2002) has been considered of very good quality by SCF, but the result from Qualichem indicates only an average level of confidence. The study by Stump (2009) was rejected by EFSA, but the Qualichem results also indicate an average level of confidence for the Qualichem respondents. Despite average levels of confidence for both studies, one was considered of good quality whereas the other was considered inconclusive. Comparison between criteria used by official expert committees and Qualichem criteria showed that SCF, ECB and EFSA seemed to prioritize certain criteria over others. However, as weighting was neither transparent nor explicit, it is difficult to assess the relative weight of different criteria, which can give an impression of subjectivity and even bias.

As a structured and transparent way of reporting study quality assessment, Qualichem has the potential to reinforce trust in safety agencies by limiting subjectivity and transparently displaying the experts' choices and assumptions. Furthermore, it makes inter-agency comparison of quality assessments of the same studies possible, by always applying the same set of methods and quality criteria.

## Supporting Information

Text S1 **Typology of quality criteria.**
(DOC)

Text S2 **Definitions of quality criteria.**
(DOC)

Text S3 **Analysis of arguments provided with the scores for controversial criteria: Tyl et al. (2002).**
(DOC)

Text S4 **Analysis of arguments provided with the scores for controversial criteria: Stump (2009).**
(DOC)

Text S5 **Expert profile.**
(DOC)

Text S6 **Comparison between quality criteria addressed in Qualichem in vivo and quality criteria addressed in other sources.**
(DOC)

Text S7 **Scale for quality assessment.**
(DOC)

Text S8 **Spreadsheet for Tyl et al. (2002).**
(XLS)

Text S9 **Spreadsheet for Stump (2009).**
(XLS)

Text S10 **Spreadsheet for Tyl et al. (2002), scores of two endocrinologists.**
(XLS)
