# Supplementary material for: Qualichem In Vivo: A Tool for Assessing the Quality of In Vivo Studies and Its Application for Bisphenol A
Source: PLoS One. 2014 Jan 29;9(1):e87738. doi: 10.1371/journal.pone.0087738 (PMC3906223; doi:10.1371/journal.pone.0087738)
Supplement: Text S1 — Typology of quality criteria. (DOC) [file pone.0087738.s001.doc]

Text S1, Qualichem in vivo: A tool for assessing the quality of in vivo studies and its application for Bisphenol A

**EXPERIMENTAL PROTOCOL (1)**

**Choice of test species / strains / sex (e.g., sensitivity)**

**Check of the properties of the substance and its formulations (e.g.: homogeneity, stability), before and during the experiment**

**Choice of the control (positive/active or negative/inactive)**

**Monitoring and reporting of the experimental animals’ parameters (age, weight, state of health, environmental conditions including temperature, light and humidity, etc.)**

**Correspondence between the characteristics of tested animals and the characteristics of exposed humans**

**(e.g., age, reproductive state, etc.)**

**Substance**

**Experimental animals**

**Monitoring and reporting of the state of control group(s) at the beginning and the end of the experiment**

**Check of the storage conditions of the substance (or of its formulations used in the experiment)**

**Assay**

**Sensitivity of the assay (ability to grasp the studied effects)**

**Choice of the experimental unit (e.g., number of animals tested simultaneously/per group)**

**Scientific robustness of regulatory guidelines (if used)**

**Number of tested groups**

**Number of control groups**

**Handling of experimental animals during the experiment**

**Procedure for obtaining formulation(s) of the substance (e.g., dilution, mix with solid diet, etc.)**

**Choice to test a single substance or a mixture**

**EXPERIMENTAL PROTOCOL (2)**

**Route of administration (e.g., to animals), compared to real (e.g., humans’) routes of exposure**

**Choice of the parameters (endpoints) for the effects to be observed**

**Choice of the exposure duration, timing (window) and frequency compared to the real exposures**

**Choice of the observation time, duration and frequency compared to the real potential time range of the effects**

**Choice of the level of the dose tested**

**Choice of the number of exposure levels (doses) tested**

**Tested exposure**

**Measured effects**

**Precision of the exposure measurement (or analytical) instruments and methods (e.g., LD, LQ)**

**Choice of the biological level observed**

**(e.g., inter-individual, individual organism, tissue, cell, biochemical, molecular)**

**Control of confounders:**

**demonstration that the tested animals are really exposed to the substance of interest, to the level of interest and are not influenced by other factors (potentially influencing the effects observed)**

**Toxicokinetic stage chosen for measuring exposure (food, blood, urine, etc.)**

**Laboratory procedures and human factors**

**Consideration given to subjective bias:**

**minimizing experimenter’s bias through simple or/and double blinding, randomization in allocating animals to groups, inter-observer reliability**

**Precision of the effect measurement instruments and methods (e.g., visual observation, microscope, etc.)**

**RESULTS**

**Results reporting**

**Results interpretation: Expert judgment**

**Results check**

**Status of peer-review**

**Concordance between the interpretation of the results (i.e., in terms of level of evidence and conclusiveness) and the raw data**

**Results analysis, reporting, interpretation and check**

**Causal interpretation**

**Interpretation of the dose – response relationship**

**Interpretation of the biological mechanism / biological significance of the findings**

**Reporting and analysis of natural / unexplained variability**

**Results analysis**

**Choice of the statistical method used for analyzing the study results**

**Statistical power**

**Analysis of errors, uncertainty and of study limitations**

**General level of theoretical understanding of the substance, its fate in the body, its biological effects, the relevant biological mechanisms of action of and generally its toxicology**

**Analysis of assumptions (e.g., that replace missing knowledge in toxicology, missing data, etc.)**

**Coherence with other studies**

**Interpretation of the relevance of animal data for humans**

**Results reporting: right form, complete, easy to understand, reporting of the relevant experimental conditions**

**Result interpretation: Epistemological context**

**Graphical representation of data and its adequacy**

**Choice of the statistical unit**

**Treatment of data before statistical analysis**

**The abstract is in accordance with the text of the paper**

**Interpretation of the functional relevance (as an “effect”) of behavioral, morphological, histological, molecular or biochemical changes**
