# Supplementary material for: Qualichem In Vivo: A Tool for Assessing the Quality of In Vivo Studies and Its Application for Bisphenol A
Source: PLoS One. 2014 Jan 29;9(1):e87738. doi: 10.1371/journal.pone.0087738 (PMC3906223; doi:10.1371/journal.pone.0087738)
Supplement: Text S2 — Definitions of quality criteria. (DOC) [file pone.0087738.s002.doc]

Text S2, Qualichem in vivo: A tool for assessing the quality of in vivo studies and its application for Bisphenol A

**EXPERIMENTAL PROTOCOL**

**Substance**

**Check of the properties of the substance and its formulations (e.g.: homogeneity, stability), before and during the experiment**

This quality criterion is related to whether the tested substance’s properties were checked before and during the experiment. If not, this could cast doubt on whether the tested targets were exposed to the intended substance rather than, for example, to some degradation products, and therefore on whether the observed effects are really due to the substance and not to impurities (for details, see [1]).

**Check of the storage conditions of the substance (or of its formulations used in the experiment)**

According to [1], defaults in sample storage can lead to either loss of analyte (through decomposition, evaporation or adsorption) or to a gain of analyte (through contamination). Both are sources of bias that can cast doubt on whether animals are really exposed to the substance intended.

**Procedure for obtaining formulation(s) of the substance (e.g., dilution, mix with solid diet, etc.)**

Mixing the tested substance into a formulation can be a source of heterogeneous exposure through the preparation. Whether the final preparation provides the intended exposure distribution in time will depend on the method used for integrating the tested substance, and on the use of an appropriate vehicle (if needed).

**Choice of the control (positive / active or negative / inactive)**

This quality criterion is related to whether an incorrect control was used in the experiment, or whether no control was used when one should have been.

By definition, a reference compound (positive control) is a compound that serves as comparison for measuring the tested effects. The properties of a reference compound are well known, and it serves as calibration tool for measuring the tested effect. Tested effects can be declared “abnormal” by comparison with already-known effects in the same class (e.g., estrogenic activity) and therefore with a level of “normality” represented by the reference compound.

The positive control is a substance whose result is already known to be positive; it is used to check that nothing went wrong during the procedure. Positive controls are used to demonstrate that a response can be detected, thereby providing some quality control on the experimental methods.

Negative controls are untreated groups of animals (or vehicle-treated groups if a vehicle is used for administering the substance) that are maintained in the same conditions as the test animals, other than exposure to the substance of interest.

**Experimental animals**

**Correspondence between the characteristics of the tested animals and the characteristics of exposed humans (e.g., age, reproductive state, etc.)**

This quality criterion is related to whether the characteristics of the animals (age, reproductive state) tested were correlated with the characteristics of the humans exposed, or whether such correlation was checked.

**Choice of the test species / strains / sex (e.g., sensitivity)**

This quality criterion is related to whether the strains of laboratory animals chosen for testing the substance were (the most) sensitive to the tested substance. Some negative effects shown by animals that are more sensitive to a substance could be missed if less sensitive animals are used. This uncertainty comes from the fact that one cannot know which negative effects could have been tested (given the experimental conditions chosen) if more sensitive animals had been used.

The choice of strain is open in regulatory toxicological testing, as in toxicological academic research. The OECD guidelines may recommend preferred species, but the experimenter can make a different choice if appropriate justification is made [2].

**Handling of experimental animals during the experiment**

Handling of animals during the experiment should not produce a level of stress that could interfere with the results. Some indications of good practice are provided in, for example, the *Guide for the Care and Use of Laboratory Animals* [3].

In particular, the effects of specific protocols (e.g., oral gavage) on experimental animals and in particular on stress are debated in the literature [4-7] but seem to depend on specific conditions (e.g., skilled, trained technician).

**Monitoring and reporting of experimental animals’ parameters (age, weight, state of health, environmental conditions including temperature, light and humidity, etc.)**

This quality criterion relates to the monitoring of the animal studied. Parameters like humidity, light or temperature can interfere with, for example, the hormonal status of the animals, and bias the experiment. Furthermore, the experimenter should be able to demonstrate that animals have been carefully identified (e.g., tagged) throughout the experiment.

**Monitoring and reporting of the state of the CONTROL group(s) at the beginning and the end of the experiment**

This quality criterion is identical for test and control groups.

**Assay**

**Sensitivity of the assay (ability to detect the studied effects)**

This quality criterion is related to whether the design of the experiment was appropriate for capturing the tested negative effects. The parameters chosen, for example, can be too rough—not specific enough and common to many causal factors—to be indicative of a particular effect.

**Choice of the experimental unit (e.g., number of animals tested simultaneously / per group)**

The results of *in vivo* studies are more precise when more animals are used. Increasing the number of animals makes it possible to discriminate between real effects and changes due to variability and chance. The size of the groups determines the statistical power of the test [2].

**Number of tested groups**

This quality criterion is directly related to the number of exposure levels (doses) tested—usually one group per dose is used. However, it is possible to make different design choices about the number of tested groups, which will influence the global quality of the study.

**Number of control groups**

This quality criterion is directly related to the choice of a positive or negative control. In experiments that include a positive control, it may be best to also include a negative control.

**Scientific robustness of regulatory guidelines (if used)**

Guidelines have been created to provide a uniform basis for regulatory evaluation of risks, despite different national contexts. Their role is as much to provide understanding of the hazards of a substance as to facilitate equal treatment of producers in different countries on the global markets, by avoiding green barriers to commerce.

Testing standards are intended to provide general information about the effects of a substance on particular targets, like reproduction or neurologic integrity. According to [8], “*the tests that are routinely carried out on regulated chemicals should detect most of the toxicological effects of a chemical, including those that occur late in life*” (pp. 34). However, “*it is not possible to predict human adverse effects with total accuracy using any test system*” (p. 32) and therefore “*it is conceivable that certain types of toxicity might not be readily detected*” (pp. 34).

Some current regulatory guidelines have been criticized because they are slow to adapt to advances in scientific knowledge and they take a conservative approach to toxicological paradigms. Traditional endpoints required in testing guidelines (e.g., weight and histopathology of endocrine organs, in the two-generation reproductive toxicity assay) might not be sensitive enough to detect specific endocrine disrupting effects [9]. In conclusion, “*on the basis of current testing requirements, there is a real potential for missing or disregarding endocrine-disrupting effects”* (pp. 100).

**Choice to test a single substance or a mixture**

When a substance is tested alone, there is potential to miss possible negative effects that appear only in mixture with another substance. If the effects of a substance were reinforced by another substance, the dose producing an effect might be incorrect. Indeed, literature shows that there may be combination effects from endocrine disrupters that each produce very small effects, and there is debate about the most appropriate way to detect the effects of real exposure situations in chemical risk assessment [10].

**Measured effects**

**Choice of the parameters (endpoints) for the effects to be observed**

This quality criterion refers to whether the effects observed in the experiment, and the parameters chosen for measuring them, are those that could be produced by the substance in real conditions. Observing inadequate endpoints can cause researchers to miss some effects [9].

**Choice of the observation time, duration and frequency compared to the real potential time range of the effects**

This quality criterion refers to whether there is a discrepancy between:

- the time to observe the effects of the tested substance in the laboratory, and the time of action of the substance in real conditions (e.g., short or medium observation time is inadequate for observing long-term chronic effects)
- the moment chosen to observe the effects and the timing of the effects (e.g., choosing inadequate post-natal day [PND] to observe certain neurobehavioral or reproductive effects in pups, in a multi-generational study)
- the frequency of the observations and the speed of the changes / effects observed (e.g., changes take place very quickly, but the time between two observations is long).

**Choice of the biological level observed (e.g., inter-individual, individual organism, tissue, cell, biochemical, molecular)**

This quality criterion is related to whether there is a discrepancy between the biological level observed in the experiment and the biological level at which the substance really acts.

**Precision of the effect measurement instruments and methods (e.g., visual observation, microscope, etc.)**

This quality criterion is related to the level of precision of the instruments and methods used for measuring the chosen endpoints.

**Tested exposure**

**Toxicokinetic stage chosen for measuring exposure (food, blood, urine, etc.)**

Exposure can be measured at different toxicokinetic levels: in the food, if work is done using external concentrations; or in blood and urine, if internal concentrations are used. The choice of the toxicokinetic level for measuring exposure might influence the final quality of the results, if it was not adapted to the particular characteristics of the substance and its toxicokinetic profile.

**Choice of the level of the dose tested**

This quality criterion focuses on whether there is agreement between the exposure tested and the corresponding real exposure. If they do not agree, conclusions observed in the laboratory would not be considered applicable to real conditions. For example, if the doses chosen are either too high or too low to represent the real exposure, or there is a too great a difference between the doses tested, this leaves room for doubt about the effects at intermediate doses.

**Choice of the exposure duration, timing (window) and frequency compared to the real exposures**

This quality criterion is related to whether there is a discrepancy between the time chosen to expose the laboratory animals to the tested substance, and the exposure time to the substance in real conditions (e.g., such an error would be to expose laboratory animals only once to a substance that humans are exposed to repeatedly. Or, laboratory animals are continuously exposed to a substance that humans are exposed to intermittently and extensively during short periods, e.g., during meals).

**Choice of the number of exposure levels (doses) tested**

This quality criterion is related to the number of levels chosen for testing the substance. Doubt about the significance of the results for real situations may persist, for example, if levels other than those tested are expected to give different effects.

**Route of administration (e.g., to animals), compared to real (e.g., human) routes of exposure**

This quality criterion is related to whether the route of exposure chosen in the laboratory is equivalent to, or representative of, the real route of exposure. For example, exposing laboratory animals to the substance by ingestion when the primary route of exposure in humans under real conditions is by contact or inhalation, or when both ingestion and contact contribute significantly to the total toxicity of the substance.

**Precision of the exposure measurement (or analytical) instruments and methods (e.g., DL, QL)**

This quality criterion is related to the precision of the analytical methods and instruments used to measure the exposure level. Problems that could affect precision include:

- use of a balance that is not well calibrated
- choosing an inadequate Detection Limit (DL) or Quantification Limit (QL) for detecting / quantifying a substance in a given matrix,
- choosing a measurement method that lacks specificity for discriminating the substance of interest from mixes of substances.

For good laboratory practice (GLP) studies, instruments are included in the internal quality assurance programme, providing insurance that they are well calibrated and correctly used. However, GLP cannot provide a scientific guarantee that the analytical method or the DL/QL are correctly chosen and appropriate for the tested substance and the specific situations of exposure.

**Control of confounders: demonstration that the tested animals are really exposed to the substance of interest, to the level of interest and are not influenced by other factors (potentially influencing the effects observed)**

This quality criterion is related to whether the researchers have demonstrated that the animals tested were really exposed to the tested substance and that they were not exposed to other disturbing factors (e.g., they are not exposed to food, water, contact or inhalation sources other than that those intentionally containing the tested substance; they are not exposed to other substances with similar endocrine disrupting effects that interfere with the tested substance, etc).

**Laboratory procedures and human factors**

**Consideration given to subjective bias: minimizing experimenter’s bias through simple or/and double blinding, randomization in allocating animals to groups, inter-observer reliability**

Voluntary or involuntary bias can influence experimenters involved in a study, especially when observations heavily demand personal skills and expert judgment [11-12]. Blinding is a solution to minimizing subjective bias.

According to [2], “*if experimenters do not know whether the animals they are collecting data from were administered a higher, lower, or no dose of the substance investigated, they will be much less vulnerable to their own expectations*.” The OECD recognizes the possibility of motivational bias, but blinding is not required in its testing guidelines. Previous studies nevertheless show that blinding could be a rather common practice in histopathology—with findings reviewed either by a second pathologist or even by a group of pathologists [2].

**RESULTS**

**Natural / unexplained variability**

**Reporting and analysis of natural / unexplained variability**

This quality criterion is related to whether variability was analyzed and reported. Variability is the uncertainty due to inherent diversity, which is especially applicable in human and natural systems, and those concerning social, economic and technological developments. Different sources of variability have been identified, such as:

- inherent randomness of nature: the chaotic and unpredictable nature of natural processes;
- technological surprise: new developments or breakthroughs in technology or unexpected consequences (side-effects) of technologies

Some examples are:

- natural variation in susceptibility to chemical toxicity or to diseases;
- natural heterogeneity between the physico-chemical composition of soils from different sites or of the water collected in different parts of a river;
- heterogeneity among individuals in parameters characterizing physiological maturation and state, e.g. pregnancy, body weight, respiratory rate and food consumption.

According to [13] “*another obvious source of uncertainty is the natural variability in time or space. The world around us is inherently variable which will also influence the risk for the protection targets. As an example: the flow rate of a river will vary between different rivers but also according to seasonal influences. It is important to note that this variability cannot be reduced by further research, it can only be characterized more accurately*”. (pp. 75)

The definition given by [8] (pp. 14) to variability refers to “*observable diversity in biological sensitivity or response, and in exposure parameters. Variability is due to inherent biological differences between species, strains, sub-strains and individuals, which cannot be reduced*.”

**Results reporting**

**Results reporting: right form, complete, easy to understand, reporting of the relevant experimental conditions**

This quality criterion is related to whether there is lack of clarity and completeness in reporting of the results that could make them more difficult to understand. It must be interpreted in the light of the publication support. Some journals still do not provide opportunities to add supplementary materials and have space constraints. Journal preference for positive results might unavoidably lead to insufficient reporting and publication bias (lower rate of publication of negative results).

**Graphical representation of data and its adequacy**

This quality criterion relates to whether graphical representations are either inadequate to efficiently communicate results (e.g., too complicated, illegible, lack of appropriate information on the two axes,) or incorrect (e.g., incorrect scale as compared to the results, use of an inappropriate graphical method).

**The abstract is in accordance with the text of the paper**

This quality criterion relates to whether the abstract agrees with the text of a study. If not, it may produce a false perception about the nature and meaning of the results.

**Results (Data) analysis**

**Choice of the statistical method for analyzing the study results**

Choice of the wrong statistical method can bias the interpretation of the raw data.

**Choice of the statistical unit**

Choice of the wrong statistical unit can bias the interpretation of the raw data.

**Treatment of data before statistical analysis**

Inadequate treatment of raw data before statistical analysis may bias the calculations and lead to an erroneous interpretation.

**Statistical power**

Statistical power is an indication about the robustness of the study results. Low statistical power or failure to calculate the statistical power may therefore cast doubt on the quality of the findings.

**Analysis of errors, uncertainty and of study limitations**

Lack of analysis of uncertainty and potential sources of error may create a biased perception about a study’s significance and lack of confidence in study authors. Self-evaluation of potential limitations of a study—in the discussion section—is recognized best practice in scientific publications.

**Results interpretation: Causal explanation**

**Interpretation of the dose-response relationship**

This quality criterion is related to assumptions about the form of the dose-response relationship, and to the use of the dose-response relationship for selecting or interpreting the study results. Significant debate exists on whether dose-response relationships are monotonic or non-monotonic for endocrine disrupters.

Dose-response is particularly important when one of the objectives of the study is to establish a NOAEL. The dose taken as the NOAEL is the highest dose below the lowest tested dose to have an adverse effect, but this is based on a monotonic dose-response profile, assuming that a lower dose will have a lower (no effect) level.

**Interpretation of the biological mechanism / biological significance of the findings**

For this criterion, lack of quality comes from the lack of scientific knowledge about the biological mechanisms triggered by the substance in the human body, or about the relevance of alternative biological explanations of the effects found. There is debate about the role of biological mechanism in interpreting toxic effects: while some argue that the fact that it is difficult to find a biological explanation should be a sufficient reason to consider findings irrelevant, others feel such findings should open the way to understand their biological underpinnings, eventually in the framework of new, mechanistic investigations.

**Interpretation of the relevance of animal data for humans**

This uncertainty is related to extrapolation from animals to humans. Depending on how close the animal model is to humans in general, on knowledge about that model as it relates to the specific effects investigated, and on the judgment of the expert about its relevancy, the significance of raw data produced in an *in vivo* study may be interpreted in different manners for different populations of interest (e.g., French pregnant women).

It is rare that animal data can be compared directly with human monitoring data. Such human data sometimes exist for poisoning or occupational exposure, but they might not be relevant for lower exposures. Some physiological differences between laboratory rodents and humans, and the difficulty of reproducing certain symptoms in animals (e.g., headache, fatigue) can be particularly problematic for studying endocrine disrupting effects.

**Interpretation of the functional relevance (as an “effect”) of behavioral, morphological, histological, molecular or biochemical changes**

Differentiating between an adverse and a non-adverse effect can be contentious in some situations. For example, [14] argues that a combination of several elements can help to establish whether an effect can be considered adverse, and if so whether there is functional relevance. An effect is considered less likely to be adverse if there is no alteration in the general function of the test organism or of the organ or tissue affected. According to [14] “*there may be effects in toxicity studies that do not represent any functional impairment in the test organism*” (p. 19). Given that changes can be found at lower (e.g., biochemical) or higher (e.g., behavioral) levels than those of the function or of the organ, the definition of an “alteration” is a matter of expert judgment. Expert judgment is sometimes highly subjective, and therefore potentially controversial.

**Epistemological**

**General level of theoretical understanding of the substance, its fate in the body, its biological effects, its relevant biological mechanisms of action, and generally its toxicology**

For this criterion, lack of quality comes from lack of knowledge, which could be reduced by further investigation [8]. Epistemic uncertainty has two inter-related origins: namely, missing data (animal studies, controlled human studies or epidemiological studies) and the limitations of the current state of scientific understanding about the topic studied (e.g., endocrine disrupters).

**Results check**

**Status of peer-review**

Though not a guarantee of absolute quality, peer-review remains a recognized method for improving the quality of scientific knowledge. Knowing whether a study has been peer-reviewed is therefore indicative of (unavoidably limited) external approval of its results.

**Coherence with other studies**

Coherence with the literature is not a guarantee of quality, and minority views in science can sometimes be more robust than majority views. However, when abundant literature starts to be published on a topic, coherence with other studies can be an indication of the relevance of the study as compared to work done by different teams.

**Results interpretation: Expert judgment**

**Concordance between interpretation of the results (i.e., in terms of level of evidence and conclusiveness) and the raw data**

Expert judgment is often used in regulatory toxicology, with or without reference-based, logical or rational argumentation. Different experts can interpret the same data very differently, and even in an opposite manner [15-16], which inevitably leads to doubt in those using expertise. Previous studies showed that considerable bias is possible in expert judgment, in relation to an expert’s personal commitment to a particular theory, his/her affiliation (industry, government or university) or his/her conflicts of interests [17-20].

**Analysis of assumptions (e.g., that replace missing knowledge in toxicology, missing data, etc.)**

When data is lacking on particular points or scientific knowledge insufficiently responds to particular demands, assumptions must be made to reach a quantitative result. Though sometimes default values or hypotheses are agreed on in the regulatory community, other times experts make subjective choices. A correct description of the results must include a description of default, case-specific and subjective assumptions [8].

**References**

1. Magnusson B, Ellison SLR (2008) Treatment of uncorrected measurement bias in uncertainty estimation for chemical measurements. Anal Bioanal Chem 390: 201-213.

2. Wandall B, Hansson SO, Rudén C (2007) Bias in toxicology. Arch Toxicol 81: 605-617.

3. National Research Council (2011) Guide for the care and use of laboratory animals. Eight edition. Available: <http://grants.nih.gov/grants/olaw/Guide-for-the-care-and-use-of-Laboratory-animals.pdf>. Accessed 17 August 2013.

# 4. Brown AP, Dinger N, Levine BS (2000) Stress produced by gavage administration in the rat. Contemp Top Lab Anim Sci 39(1): 17-21.

# 5. Turner PV, Vaughn E, Sunohara-Neilson J, Ovari J, Leri F (2012) Oral Gavage in Rats: Animal Welfare Evaluation. J Am Assoc Lab Anim Sci 51(1): 25–30.

6. Walker MK, Boberg JR, Walsh MT, Wolf V, Trujillo A, et al. (2012) A less stressful alternative to oral gavage for pharmacological and toxicological studies in mice. Toxicol Appl Pharmacol 260(1): 65-69.

# 7. Arantes-Rodrigues R, Henriques A, Pinto-Leite R, Faustino-Rocha A, Pinho-Oliveira J, et al. (2012) The effects of repeated oral gavage on the health of male CD-1 mice. Lab Anim (NY) 41(5): 129-134.

8. COT (Committee on Toxicity of Chemicals in Food, Consummer Products and the Environment) (2007) Variability and Uncertainty in Toxicology of Chemicals in Food, Consumer Products and the Environment. Available: <http://cot.food.gov.uk/cotwg/wgvut/> Accessed 17 August 2013.

9. Kortenkamp A, Martin O, Faust M, Evans R, McKinlay R, et al. (2011) State of the art assessment of endocrine disrupters. Brussels: European Commission. 135 p.

10. Kortenkamp A (2008) Low dose mixture effects of endocrine disrupters: implications for risk assessment and epidemiology. Int J Androl 31: 233-240.

11. DeMets DL (1999) Statistics and ethics in medical research. Sci Eng Ethics 5(1): 97–117.

12. Fossati R, Confalonieri C, Apolone G, Cavuto S, Garattini S (2002) Does a drug do better when it s new? Ann Oncol 13: 470-473.

13. Vermeire TG (2009) Evaluating uncertainties in an integrated approach for chemical risk assessment under REACH: more certain decisions? PhD thesis. Available: <http://igitur-archive.library.uu.nl/dissertations/2009-0602-201023/UUindex.html>. Accessed 17 August 2013.

14. ECETOC (2002) Recognition of, and differentiation between, adverse and non-adverse effects in toxicology studies. Brussels; Technical report no. 85. 60 p.

15. Rudén C (2001) Interpretations of primary carcinogenicity data in 29 trichloroethylene risk assessments. Toxicology 169: 209–225.

16. Beronius A, Rudén C, Håkansson H, Hanberg A (2010) Risk to all or none? A comparative analysis of controversies in the health risk assessment of Bisphenol A. Reprod Toxicol 29: 132-146.

17. Lynn FM (1986) The interplay of science and values in assesing and regulating environmental risks. Sci Technol Hum Val 11(2): 40–50.

18. Koehler JJ (1993) The influence of prior beliefs on scientific judgments of evidence quality. Organ Behav Hum Dec 56: 28–55.

19. Barnes DE, Bero LA (1998) Why review articles on the health effects of passive smoking reach different conclustions. JAMA 279(19): 1566–1570.

20. Maxim L, Van der Sluijs J, 2010. Expert explanations of honeybee losses in areas of extensive agriculture in France: Gaucho® compared with other supposed causal factors. Environ Res Lett 5(1): 12. Available: <http://iopscience.iop.org/1748-9326/5/1/014006>. Accessed 17 August 2013.
