# Supplementary material for: Qualichem In Vivo: A Tool for Assessing the Quality of In Vivo Studies and Its Application for Bisphenol A
Source: PLoS One. 2014 Jan 29;9(1):e87738. doi: 10.1371/journal.pone.0087738 (PMC3906223; doi:10.1371/journal.pone.0087738)
Supplement: Text S3 — Analysis of arguments provided with the scores for controversial criteria: Tyl et al. (2002). (DOC) [file pone.0087738.s003.doc]

Text S3, Qualichem in vivo: A tool for assessing the quality of in vivo studies and its application for Bisphenol A

**Analysis of arguments provided with the scores for controversial criteria: Tyl et al. (2002)**

The respondents provided written justification for why they assigned a score to each criterion, and these are presented below for the application of Qualichem to the Tyl et al. (2002) study. Our goal was to synthesize their explanations without critically commenting on them.

**Choice of the control (positive / active or negative / inactive)**

The expert respondents who assigned 6 to this criterion argued that the guideline does not demand a positive control, nor is one required even more generally in a toxicological study. One expert respondent explained that this is because guidelines are usually designed to screen effects of new substances with unknown properties when industry makes a request to authorize a new chemical. Without previous knowledge about the properties of a substance, setting a positive control would be difficult or even impossible. Furthermore, other respondents argued that BPA seems to act on humans not only through estrogenic mechanisms, but in a more complex manner that would not be captured by using an estrogenic control.

Of those expert respondents who felt there was a need for a positive control (e.g., 17-β-estradiol, ethinyl oestradiol or other known estrogen), not all placed the same importance on it. Whereas some considered it absolutely necessary to detect a reproductive effect of BPA, others noted mitigating circumstances and referred to the additional burden of including such a control group in an experiment that already involved a significant number of tested groups. One expert respondent also cited the ambiguity in the article about whether or not the vehicle (acetone) was used in control diets as a reason for assigning a low score to this criterion. This is also a demand of the OPPTS guideline: “*If a vehicle is used in administering the test substance, the control group should receive the vehicle in the highest volume used*” [1] (p. 3). As well, the guideline recommends that effects of the vehicle on absorption, distribution, metabolism, retention or toxic properties of the test substance, and more generally on animals, should be considered. Information about the volume and potential effects of the vehicle is not available in the paper.

**Choice of the test species / strains / sex (e.g., sensitivity)**

For all the expert respondents who assigned low scores to this criterion, questions arose about the sensitivity of Sprague-Dawley rats to estrogenic substances. The authors declare that they tested the sensitivity of their strain to dietary E2 and BBP, but those data are unpublished and details such as tested doses and types of effects observed are unavailable. This sustains doubt, as some published literature seems to contradict such sensitivity. Expert respondents felt information about the sensitivity of the test strain was necessary, even within the constraints associated with reduced space available in a scientific article, and noted that it is frequently an option to provide supplementary material.

Expert respondents who assigned high scores to this criterion either trusted the reference to the regulatory guideline or trusted the laboratory that performed the study, considering its availability of historical data. Also, the OPPTS guideline only specifies that strains of low fecundity should not be used.

**Monitoring and reporting of experimental animals’ parameters (age, weight, state of health, environmental conditions including temperature, light and humidity, etc.)**

Most expert respondents were satisfied with reference to the guideline and the description given by the authors. Scores of 5 were given by those expert respondents who were unable to find reference in the paper to the age of the animals when they were received by the lab. The OPPTS guideline [1] recommends an age of 5 to 9 weeks, but one expert respondent considered that the difference in the hormonal status of the animals at 5 weeks compared to 9 weeks can be quite important—by 9 weeks they are more sexually mature and are potentially eating less than at 5 weeks. For this reason, such information would have been useful in the article.

The most critical expert respondent referred to the temperature (64 – 79°F) and the relative humidity (30 – 70%) during the experiment. As temperature influences hormone levels in animals, its broad range might have had consequences on the experiment. The OPPTS guideline does not contain direct specific recommendations about these two parameters; rather, it refers to a guideline for the care and use of laboratory animals. However, the OECD 416 guideline recommends a temperature range of 22±3°C (71.6°F, range from 66.2 – 77°F). The range declared in the paper is too broad when compared to the OECD guideline. OECD recommends at least 30% humidity, and preferably not more than 70% except during room cleaning—the aim is 50 – 60%.

**Sensitivity of the assay (ability to capture the studied effects)**

Several expert respondents argued that the design of the assay does not allow for in-depth investigation of BPA reprotoxic effects. They summarize their criticism about other criteria as either related to exposure (continuous) or effects (too short time of observation during adulthood, lack of observation on fine parameters indicative of estrogenic effects). Criticism about the parameters used for measuring the reprotoxic effects was most common, with those actually used in the study considered too rough. Lack of precision in the description of methods for measuring some parameters (e.g., sperm counts, histopathological) reinforced doubt.

**Number of control groups**

Only one expert respondent assigned a score of 1 for this criterion, consistent with the criticism about the lack of a positive control. In fact, this expert argued the study needed two control groups: one positive and one negative.

**Scientific robustness of regulatory guidelines**

Criticism focused on the regulatory guidelines that were directly related to the elements highlighted in relation to the sensitivity of the assay, and in particular to the parameters considered too rough to capture reprotoxic effects of BPA. Though such guidelines are considered appropriate for screening the effects of new substances, several expert respondents considered them inappropriate when knowledge already exists about a substance. Furthermore, the guidelines for investigating reprotoxic effects are judged too old, and therefore do not adequately integrate the current scientific knowledge. For this reason, they do not respond to many situations of exposure and kind of effects encountered with endocrine disrupters. Examination of additional parameters, like nipple/areolae retention in preweanling males, was seen as recognition of the limitations of the guidelines by the authors themselves, though the parameters measured were still seen as insufficient subset of the range of potential effects.

### It should be highlighted here that some expert respondents confused the OPPTS and OECD guidelines [1-2]. At least three of them were convinced at the beginning of our interview that the study follows an OECD guideline. The image of “guidelines” (in general) as unquestionable references of quality is so strong that respondents did not even check which guideline was relevant to this particular study. A relevant anecdotal detail is that the study authors made a small error, and cited the OPPTS 837.3800 rather than the OPPTS 870.3800 guideline in both in the abstract and the paper, though the reference list contains the correct number. None of the expert respondents we interviewed noticed this error and all trusted the authors’ reference to a guideline. Nevertheless, there is no 837 guideline**[[1]](#footnote-2)**. Also, some expert respondents were convinced that the study had followed an OECD guideline, and were astonished to find that only an OPPTS guideline had been cited in the study. Only one expert respondent referred to their own experience of studies that claimed to respect guidelines even though they did not respect all the requirements.

**Choice of the parameters (endpoints) for the effects to be observed**

This was the most critical aspect of the study for many expert respondents, significantly driving the lack of sensitivity of the design. Missing parameters include cancerous transformations of the mammary gland, polycystic ovary syndrome, metabolic syndrome, and effects on development and the nervous and immune systems. Furthermore, the thyroid has important roles in growth, brain development and female reproduction, but was not included. Ano-genital distance was not measured in F1offspring, but only in F2 and F3, which is in accordance with the OPPTS guideline but not with an objective to address relevant endpoints highlighted in the scientific literature.

Another element that raised doubts refers to the number of animals used for hystopathological comparison, i.e., 30 for controls and 10 for each dose group. One expert respondent considered histopathology as the most relevant level where effects can be seen, and argued that even if recommended by the guideline, 10 animals per group is too small to provide enough statistical power and is particularly imbalanced compared to 30 animals in the controls.

Some expert respondents had a problem with lack of raw data for a parameter indicative of fertility effects, i.e., the numbers of females getting pregnant (the mating rate of success) from the total number of females mated.

Finally, some parameters lack clear description (e.g., gestational index) and that prevented respondents from forming a confident opinion about them.

**Choice of the observation time, duration and frequency compared to the real potential time range of the effects**

One of the most common critiques was that the adult observation period was too short. Indeed, some effects could appear into adulthood, whereas the rats in the experiment represented rather young individuals. Three individuals per sex and per generation were necropsied immediately after weaning, all F0, F1, F2 and F3 males and were necropsied at delivery and females at weaning of their progeny. However, effects like cancer or anovulatory syndrome may appear later in life.

Expert respondents directed this critique at the regulatory guideline, which is supposed to demand this observation time. Nevertheless, a close look shows that the guideline does not specify the exact time to terminate animals and leaves it to the study authors to decide how long to observe reproductive effects: “*All P and F1 adult males and females should be terminated when they are no longer needed for assessment of reproductive effects*” [1] (p. 6). The observation timing was based on the authors’ interpretation of the guideline, rather than a strict regulatory requirement.

One expert respondent assigned a score of 5, arguing that the timing for measuring ano-genital distance was not appropriate for providing accurate measurements. Pups are very small and their movements difficult to control at post-natal day (PND) 0, so there may have been significant measurement error.

**Choice of the biological level observed (e.g., inter-individual, individual organism, tissue, cell, biochemical, molecular)**

Two expert respondents assigned a score of 5 to this criterion, arguing that measuring the level of BPA in the blood would have helped to better understand the (lack of) effects.

More critical expert respondents argued that measurements at molecular or biochemical levels should have been used to analyze the relevance of gross findings (including a check of lack of effects on rough parameters). Some effects might have been missed because the level of observation was too aggregate and not enough attention was paid to cellular effects in mammary glands or receptors in the brain.

**Choice of the exposure duration, timing (window) and frequency compared to the real exposures**

Only three of the expert respondents considered continuous exposure as relevant to humans—all five others questioned this. According to one expert respondent, exposing animals over all three generations does not allow for observation of trans-generational effects—exposure of one generation and observation of effects in another, non-exposed generation. With such a continuous exposure, it would not be possible to know where the effects come from and when they appear. Furthermore, gavage would be a better comparison to the pattern of human eating, which is not continuous as for rats fed *ad libidum*–even more so because the patterns of effects seem to be different in short-term, repeated exposure versus continuous exposure.

Continuous exposure would also not represent vulnerable populations like formula-fed infants. As BPA is not highly fat-soluble, one respondent argued that exposure through milk might in fact be very low or non-existent. More generally, the early post-natal period is not represented or at least there is no precise information about the reality and the levels of exposure of pups. Another category that is not represented in the study is adults, as F0, F1, F2 and F3 were all necropsied after delivery or after weaning. However, humans continue to be exposed throughout their life, and such life-long exposure is not captured by the time of exposure of the rats.

The OPPTS and OCDE guidelines do not restrict the pattern of exposure to either continuous or gavage, and leave this choice to the study authors.

**Choice of the level of the dose tested**

Most expert respondents referred here to the interval between the lowest and the highest dose, and considered this generally fine and inclusive of all potential exposures in humans. One expert respondent was very critical of the low number of low doses, suggesting that more low doses would have been needed because they are closer to human exposure than the higher doses tested. Furthermore, there would be a dramatic difference between the different dose levels, with factors of 15 to 20 between 0.015, 0.3, 4.5 and 75, whereas the guideline recommends two- to four-fold factors and avoiding factors above 10. The OECD guideline even specifies: “*For the dietary studies the dose interval should not be more than 3 fold*” [2] (p. 3).

Because of the significant variability in ingested doses of BPA found in the study, another expert respondent moderated their answer to a 5, considering that it is difficult to imagine the consequences of such important changes in exposure, like from below the NOAEL to more than three times the NOAEL in one group.

**Control of confounders: demonstration that the tested animals are really exposed to the substance of interest, to the level of interest and are not influenced by other factors (potentially influencing the effects observed)**

One of the criticisms related to this criterion was that phytoestrogens were present in the food administered to the rats at rather high levels, and were potentially interfering with the action of BPA. The argument that potential effects could have been seen in controls can be refuted with the argument that, if tested groups were already exposed to these confounders, the “zero” point of departure for the estrogenic effects of BPA would already be higher than thought.

In addition, the study lacked precision about contaminants in the water, which the authors claim to be found below the maximum levels defined for drinking water. They do not specify limits or which substances they tested for. Another respondent raised the question of the background level of BPA (for example, in the air) but did not adjust the score they assigned based on this, because it is current practice to ignore such aspects in toxicological work.

**Consideration given to subjective bias: minimizing experimenter’s bias through simple or/and double blinding, randomization in allocating animals to groups, inter-observer reliability**

All criticism relating to this criterion focused on the lack of blinding. However, half of the respondents to this question considered that blinding is not absolutely necessary for the gross measurements made in the study, can be difficult to put in practice, or are not widely practiced in toxicological experimentations (or, on the contrary, probably are current practice in the laboratory but not reported in the article).

The other half of the expert respondents argued that some of the measurements for which undisputable observational rules do not exist—especially on histopathology, ano-genital distance and sperm analysis—might have been influenced by the experimenter, and blinding would have been better.

**Graphical representation of data and its adequacy**

Most expert respondents felt graphical representations were correct and clear. Two of them, to different extents, felt the graphical representations are too complicated to be helpful.

**The abstract is in accordance with the text of the paper**

One expert respondent was very critical about the authors’ interpretation of their raw data, and considered it selective. This expert suggested that the authors chose to ignore elements that could have indicated effects and failed to recognize limitations, making the study too positive. As this interpretation is also reflected in the study’s abstract, this expert assigned a minimal score for this criterion.

**Treatment of data before statistical analysis**

The authors determined that some data lacked biological relevancy and therefore excluded it from statistical analysis. Though this did not concern most expert respondents, one was very critical about the method through which some data was considered biologically implausible. Given the state of knowledge on BPA, this expert respondent argued that the authors should have attempted to understand all data, or at least recognize the limitations in current understanding. Invoking lack of biological relevance was seen here as an unfounded and scientifically illegitimate manner of excluding data that might have put into question the general argument of the paper.

As well, pooling data from the different generations should have been done only after a careful check of any potential differences between the groups. The way this was tested and described in the paper was found to be imprecise, especially regarding the conclusion that there was “*no statistical differences in the distribution of ages among groups*” [3] (p. 139).

**Statistical power**

Most respondents felt that the high number of animals and litters gave the study good statistical power. However, this sample size was rather a secondary effect of the study design than a planned component. The most critical respondent argued that statistical power should have been calculated before the beginning of the study and the number of animals should have been adapted to that calculation. Probably, fewer animals could have been used with the same statistical power. One respondent gave a score of 3, noting that it is not clear why a histopathological comparison was done between 30 animals for controls and only 10 animals for tested groups, which probably reduced statistical power.

**Interpretation of the dose-response relationship**

Whether the dose-response relationship is monotonic or non-monotonic is central in the debate on endocrine disrupters in general and BPA in particular. For this reason, establishing whether a non-monotonic dose-response curve exists was an explicit objective in the Tyl et al. study. This attempt to characterize a dose-response relationship was considered legitimate and received a maximal score from half of the respondents. One respondent that assigned a score of 6 did note that it is difficult to understand how calculations were done and the details of the General Linear Models or nonparametric tests used in the study. The more critical expert respondents felt that the report was missing information about these calculations that would have helped facilitate understanding of what exactly had been done. Two of them expressed an impression that dose-response had been used too easily to justify the exclusion of effects that were found to be significantly related to BPA, instead of discussing whether it is plausible that these findings were real effects.

**Interpretation of the biological mechanism / biological significance of the findings**

Two respondents were very critical (scores of 1) about how the study authors dealt with biological mechanisms. For example, they felt that it was inappropriate scientific practice to exclude significant findings on ano-genital distance (AGD) by considering them biologically irrelevant. One respondent argued that the authors could not know in advance what distance can be considered biologically relevant. Therefore, the argument that the increase in AGD in F2 females was “minimal” should not have been used to exclude data that might have actually been an effect of BPA. Those expert respondents who assigned scores of 6 argued that the objective of the study was not to understand biological mechanisms, and so the authors’ treatment of AGD was in accordance with their objective.

**Interpretation of the relevance of animal data for humans**

The paper makes only one explicit reference to what study findings can be extrapolated to effects for humans. A note below a table refers to an animal with developmental malformations, which were interpreted as being the result of a spontaneous genetic mutation that also exists in humans. One expert respondent approved of this interpretation and assigned a score of 6, while another assigned a score of 5, referring to the authors’ failure to try to discuss how their findings might be significant for humans. The most critical expert respondent (score of 1) argued that the study design was not relevant for human exposure because it did not test enough low doses and the dose ranges were too broad.

**Concordance between interpretation of the results (i.e., in terms of level of evidence and conclusiveness) and the raw data**

Four of the respondents found the interpretation of the results was in accordance with raw data. Two were more skeptical, giving scores of 2 and 4 based on the criticism that the raw data show significant effects at lower doses than the NOAEL established by the authors (750 ppm), but the authors considered this irrelevant. These two respondents have doubt about whether the raw data allow the authors to make the conclusions they have made.

**Status of peer-review**

Peer-review is considered by many respondents as a good, scientifically accepted means of checking results, even if it cannot be considered as a perfect token of scientific quality. The quality of studies will depend on the journals and on the reviewers. *Toxicological Sciences* is considered a good journal, so many respondents gave a score of 6 to this criterion. Only two were very critical (scores of 1 and 2), arguing that a journal in another disciplinary background (endocrinology) would have not accepted such a paper.

Of the 26 critical criteria, five had a median under 4 (in the orange or red zones):

**Toxicokinetic stage chosen for measuring exposure (food, blood, urine, etc.)**

Most expert respondents felt that exposure should have been confirmed somehow, notably through an internal measurement in blood. Without such measurement, there remains significant doubt about whether animals were really exposed to BPA, and if so, what were the real levels of exposure. Some respondents also said they had difficulty understanding the calculation of exposure because of lack of precision in the method of calculating the actual intake.

Regarding internal measurements, the OPPST guideline requires that: “*In any study that demonstrates an absence of toxic effects, further investigation to establish absorption and bioavailability of the test substance should be considered*” [1] (p. 9) This specification was not considered by the study authors.

**Analysis of errors, uncertainty and study limitations**

Only two expert respondents responded to this question, and they provided opposite assessments (1 and 6). All others considered themselves not competent or unable to respond (”cannot answer”). The critical respondent argued that the authors were too affirmative about their work, and failed to follow the good scientific practice of highlighting possible limitations of one’s work.

**Analysis of assumptions (e.g., that replace missing knowledge in toxicology, missing data, etc.)**

The body weight and daily feed consumption for males and females, used in the calculation of the daily BPA intake, are explicit assumptions in this study. The most critical respondents felt that many other assumptions are implicitly contained at many levels of the work, from setting up the study design (e.g., establishing the parameters to be observed) to interpretation of the results (e.g. what is considered biologically relevant, how dose-response was considered). Low scores for this criterion were attributed to the lack of explicit discussion of these assumptions and concern that the assumptions were not plausible. Interestingly, three expert respondents were unable to identify implicit assumptions and even skipped the explicit assumptions presented below a table.

**Coherence with other studies**

Coherence with other studies is not necessarily an indisputable criterion for the quality of a study. Minority views or unique expertise on specific subjects can actually be the best available scientific knowledge. This criterion was added only in the second half of our work, so only four experts were invited to respond. Among them, two respondents did not feel the question was applicable, because a similar design had not been done before or the literature quoted in the study had been selective. One respondent gave a score of 6, arguing that the results are in accordance with some of the literature on BPA. One expert respondent gave a score of 2, arguing that most of the literature shows reproductive effects at doses lower than the NOAEL established in this study.

**General level of theoretical understanding of the substance, its fate in the body, its biological effects, its relevant biological mechanisms of action, and generally its toxicology**

The responses to this question ranged from 1 to 6, but half of the respondents gave a minimal score. They argued that the current state of knowledge does not allow for a good understanding of the toxicological profile of BPA, despite the many studies available. In assessing the current state of knowledge, respondents particularly considered the quality of the existing studies.

**References**

1. U.S. EPA (1998) Health Effects Test Guidelines, OPPTS 870.3800, Reproduction and Fertility Effects (Final Guideline, August 1998). Washington, DC: EPA. Available: <http://www.epa.gov/oppts/pubs/frs/publications/Test_Guidelines/series870.htm>. Accessed 10 August 2013.

# 2. OECD (2001) OECD Guidelines for the Testing of Chemicals. Test No. 416: Two-Generation Reproduction Toxicity. Paris: OECD Environment Directorate. Available: [**http://oberon.sourceoecd.org/vl=692122/cl=21/nw=1/rpsv/ij/oecdjournals/1607310x/v1n4/s26/p1**](http://oberon.sourceoecd.org/vl=692122/cl=21/nw=1/rpsv/ij/oecdjournals/1607310x/v1n4/s26/p1) Accessed 10 August 2013.

3. Tyl RW, Myer CB, Marr MC, Thomas BF, Keimowitz AR, et al. (2002) Three-Generation Reproductive Toxicity Study of Dietary Bisphenol A in CD Sprague-Dawley Rats. Toxicol Sci 68: 121-146.

1. This errors should be corrected, as the same reference to a non-existent OPPTS 837.3800 guideline can be found on the ECHA website for another substance, 4-tert-butylphenol. [↑](#footnote-ref-2)
