# Supplementary material for: Qualichem In Vivo: A Tool for Assessing the Quality of In Vivo Studies and Its Application for Bisphenol A
Source: PLoS One. 2014 Jan 29;9(1):e87738. doi: 10.1371/journal.pone.0087738 (PMC3906223; doi:10.1371/journal.pone.0087738)
Supplement: Text S4 — Analysis of arguments provided with the scores for controversial criteria: Stump (2009). (DOC) [file pone.0087738.s004.doc]

Text S4, Qualichem in vivo: A tool for assessing the quality of in vivo studies and its application for Bisphenol A

**Analysis of arguments provided with the scores for controversial criteria: Stump (2009)**

The respondents provided written justification for why they assigned a score to each criterion, and these are presented below for the application of Qualichem to the Stump (2009) study. Our goal was to synthesize the respondents’ explanations without critically commenting on them.

**Check of the storage conditions of the substance (or of its formulations used in the experiment)**

One respondent gave a score of 1 to this question, without giving further details, while the remaining three respondents gave a score of 6.

**Sensitivity of the assay (ability to detect the studied effects)**

Two respondents assigned a score of 6 for this criterion: one did not provide further explanation and the second cited respect of a regulatory guideline—representing an “international consensus” between governments—as evidence of the study’s quality. One expert respondent assigned a score of 5, citing the surprising lack of male-female differences for one of the parameters measured and for high variability in other results. The fourth and most critical expert respondent assigned a score of 2, also noting that male-female differences should have been found in learning and memory and that the variability in the behavioral testing results was too high. Given the low score, this expert respondent more heavily weighted the importance of these two problems, and considered them a critical indication that the assay was not sensitive enough.

**Scientific robustness of regulatory guidelines**

This criterion received quite polarized scores. Two expert respondents assigned a score of 6 and the other two assigned a score of 2. One of the two expert respondents who assigned a maximal score argued that the importance is not in the guideline itself, but how it was applied. For substances for which any knowledge exists, the guideline should be able to screen some rough effects. The guideline was considered less relevant for substances on which a lot of knowledge already exists. Furthermore, it is recognized that a guideline is not “frozen” in its demands, as it allows for many possible ways to adapt its requirements.

The critical respondents argued that the OECD guideline was either incomplete or too vague about exactly how the recommended methods had to be applied to be useful. For these reasons, the guideline was helpful neither for the experimenter nor for the regulator.

**Choice of the parameters (endpoints) for the effects to be observed**

One expert respondent assigned a score of 6 to this criterion, adding that reference to an international guideline is adequate for ensuring a study’s quality. This expert respondent also had previous experience with the work of the laboratory that performed the study and knowledge of the people who conducted it.

Two other expert respondents assigned lower scores of 5 and 3, citing slight to moderate criticism that the range of parameters investigated was not exhaustive; for example, other forms of learning could have been tested because the Biel maze is insufficiently sensitive. Exclusion of these parameters was only partly justified by the current incomplete state of knowledge about neurobehavioral toxicity in mammals.

The most critical expert respondent (score of 1) felt that other parameters definitely should have been studied. Guidelines only represent a starting point that can be adapted for substances for which some knowledge exists, but not for well-studied substances like BPA. Previous studies on BPA indicate potential effects on anxiety or on sexual dimorphic behavior that were not addressed by the Stump study. In that sense, because there is common misunderstanding that a guideline study is the best possible study, previous scientific knowledge on BPA was ignored when the study design was drafted. Such parameters would have been justified, as the OPPTS 870.6300 guideline recommends “*the test of learning and memory be chosen on the basis of its demonstrated sensitivity to the class of compound under investigation, if such information is available in the literature. In the absence of such information, examples of tests that could be made to meet the above criteria include: delayed-matching-to-position (...), olfactory conditioning (...) and acquisition and retention of schedule-controlled behavior*” [1] (p. 5). The OECD guideline makes similar recommendations.

In the Stump study, there was no reference to whether or not literature exists that addresses how well the Biel maze or other tests capture the kind of effects on learning and memory expected from endocrine disrupters like BPA. Furthermore, there were no arguments about how the performance of the Biel maze compares to other possible tests.

Other details from the guidelines were also modified by the authors:

- neuropathological examinations were done on post-natal day (PND) 21 instead of PND 11 as recommended by the OPPTS guideline [1]. On this point, the two guidelines used by the authors do not agree, as the OECD guideline recommends neuropathological evaluation at PND 22 or at some point between PND 11 and PND 22.
- all animals were killed by intraperitoneal injection instead of exposure to carbon dioxide [1]
- the study does not describe how neuropathological analysis of the animals was done—EPA recommends following the OPPTS 870.6200
- brains were weighed after at least 36 hours in fixative instead of immediately after killing the animal (OPPTS and OECD guidelines)

None of the expert respondents interviewed addressed these differences—it is not clear whether any of them carefully compared the EPA / OECD guidelines with the Stump study to check for compliance, or if they simply “trusted” the statements of the authors.

**Precision of the effect measurement instruments and methods (e.g., visual observation, microscope, etc.)**

The responses for this criterion were similar to those provided for “choice of parameters”. One expert respondent gave a score of 6, citing confidence that following the OECD guideline is an indication of quality, and that he knew the people who performed the study. All others gave scores ranging between 1 and 4. The critical expert respondents noted that other, more precise methods exist and could have been used. The most critical expert respondent suggested that the variability was too high for the methods to have been precise enough. One of the expert respondents addressed the issue of “censored data[[1]](#footnote-2)” cited in the EFSA evaluation of this study—this expert respondent questioned the view of EFSA on this point and suggested that EFSA statisticians were incorrect.

**Toxicokinetic stage chosen for measuring exposure (food, blood, urine, etc.)**

Only one expert respondent gave a score lower than 6 on this point—this expert suggested that some internal measurement of BPA should have been done to check the exposure, especially in pups. If exposure is insufficient during the lactation period, according to the OECD guideline, pups might have been dosed directly: “*Direct dosing of pups should be considered based on exposure and pharmacokinetic information (...). Careful consideration of benefits and disadvantages should be made prior to conducting direct dosing studies*.” [2] (p. 4-5). Exposure of pups is of particular importance because of the difference between rats and humans: rat pups are born in an earlier stage of development than are humans—part of rat brain development happens after birth. This would correspond to the last trimester of fetal life in humans. Therefore, if some kind of check for exposure through milk is not used to confirm that pups are actually exposed to BPA, no firm conclusion can be expressed about the influence of BPA on the brain development of these pups or, by generalization, on BPA neurotoxicity. Therefore, it is not possible to use these data to do a risk assessment for the last trimester of human fetal development.

Two other expert respondents made the same comment, but considered the concern insufficient to give a score lower than 6, arguing that such measurement is not demanded in the regulatory guidelines, and that risk assessment would be always done based on external exposures.

**Choice of the statistical method for analyzing the study results**

One expert respondent gave a score of 6, arguing that the authors performed the usual statistical tests. One expert respondent gave a score of 5, arguing that their own practice and that of statistician colleagues show that EFSA statisticians were putting too much importance on the issue of censoring. According to this respondent, statisticians usually disagree anyway, so it is difficult to say what “best scientific practice” means. The most critical respondent assigned a score of 2, and agreed with EFSA statisticians that different statistical methods should apply to censored data. This expert respondent also argued that high variability in the results also indicated problems with the statistical method applied (score of 2). The fourth respondent could not answer based on a lack of specific competence in statistics.

**Statistical power**

Again, three respondents provided a score. Only one was heavily critical of the study on this point, assigning a score of 1 based again on the high variability in certain results. Another expert respondent argued that statistical power was acceptable as the number of animals was large enough. The third did not offer an explanation for their score; however, this expert respondent’s general line of argumentation for most of the answers was to reference adherence to the regulatory guideline and knowledge of the team having performed the study.

**Analysis of errors, uncertainty and of study limitations**

Two respondents suggested that the study does not adequately represent the variability of its results and potential errors. They noted that best scientific practice always demands an analysis of limitations and potential sources of error in any study. Two others assigned a score of 6, noting that such an analysis was not needed.

**Interpretation of the biological mechanism / biological significance of the findings**

The critical responses to this question directly related to how dose-response was addressed in the study. Two expert respondents suggested that searching for a plausible biological explanation would have been an appropriate way of interpreting significant findings, rather than excluding them based on lack of dose-response.

**Concordance between interpretation of the results (i.e., in terms of level of evidence and conclusiveness) and the raw data**

One of the respondents assigned a score of 1, and suggested that the authors made an incorrect interpretation of the effects found at low dose from their raw data. Statistically significant findings were discarded in the study based on the lack of dose-response relationship; however, this respondent felt that these results could indicate an effect or they could be the result of natural variability. Completely excluding the effects in this case does not allow for discussion about whether these were real effects or random findings, and from this point of view the authors were considered to be much too affirmative.

A second expert respondent gave a score of 5 on this criterion, arguing that the authors somewhat over-interpreted the data; given that the parameters chosen for measuring neurotoxicity were rather rough, the conclusion drawn by the authors, referring to “developmental neurotoxicity”, seemed overstated.

**Analysis of assumptions (e.g., that replace missing knowledge in toxicology, missing data, etc.)**

Two of the three expert respondents who answered this question suggested that the authors did not disclose or analyze hidden assumptions, as they should have in accordance with best scientific practices, and that this influenced the results of the study,

**Reporting and analysis of natural / unexplained variability**

One expert respondent assigned a score of 6 to this criterion, arguing that standard deviation is the usual way of representing variability. Another expert respondent argued that variability would have been better represented after a lognormal transformation of the data. The most critical respondent argued that there was no real analysis of the important variability found for some results. This analysis would have provided a better understanding of the ability of the study design to capture the intended effects, by showing which parts of the variability were natural and which were due to the method itself.

**Interpretation of the dose-response relationship**

Two expert respondents were highly and moderately critical with respect to this criterion, referring essentially to the fact that significant statistical effects should not be excluded based on a lack of dose-response, particularly because some effects could follow non-monotonic dose-response relationships.

**General level of theoretical understanding of the substance, its fate in the body, its biological effects, its relevant biological mechanisms of action, and generally its toxicology**

This criterion had the highest aggregated uncertainty (the lowest median), with scores ranging from 2 to 6. The expert respondent who assigned the maximal score referenced the public debate on BPA, which would make the effects of BPA look misleadingly important. The other three expert respondents assigned scores of 2 or 3, and provided little explanation.

**References**

1. U.S. EPA (1998) Health Effects Test Guidelines: OPPTS 870.6300, Developmental Neurotoxicity Study. EPA 712-C-98–239. August 1998. Washington, DC: EPA. Available: <http://www.epa.gov/oppts/pubs/frs/publications/Test_Guidelines/series870.htm>. Accessed 10 August 2013.

2. OECD (2007) OECD Guidelines for the Testing of Chemicals. Test No. 426: Developmental Neurotoxicity Study. Paris: OECD Environment Directorate. Available: <http://oberon.sourceoecd.org/vl=692122/cl=21/nw=1/rpsv/ij/oecdjournals/1607310x/v1n4/s26/p1> Accessed 10 August 2013

3. EFSA (2010) Scientific Opinion on Bisphenol A: evaluation of a study investigating its neurodevelopmental toxicity, review of recent scientific literature on its toxicity and advice on the Danish risk assessment of Bisphenol. EFSA Journal 8(9): 116.

1. EFSA gives the following definition for censoring: “In statistical terminology censoring occurs when the value of an observation is only partially known. In the Biel water maze test the number of errors made by the rats that did not complete the maze within the 3 minute limit that was used, was recorded and the time to escape was recorded as 180 seconds. These experiments are “right censored”, meaning that the rats could have escaped and made more errors, had they been given sufficient time. Therefore the time to escape and the number of errors recorded will have been underestimated.” [3] (p. 3) [↑](#footnote-ref-2)
