# Supplementary material for: Qualichem In Vivo: A Tool for Assessing the Quality of In Vivo Studies and Its Application for Bisphenol A
Source: PLoS One. 2014 Jan 29;9(1):e87738. doi: 10.1371/journal.pone.0087738 (PMC3906223; doi:10.1371/journal.pone.0087738)
Supplement: Text S5 — Expert profile. (DOC) [file pone.0087738.s005.doc]

Text S5 Qualichem in vivo: A tool for assessing the quality of in vivo studies and its application for Bisphenol A

**1. EXPERT PROFILE**

**Name** (for recording, the analysis will treat the answers anonymously):

**Institution** (for recording, the analysis will treat the answers anonymously):

**Function** (for recording, the analysis will treat the answers anonymously):

Please respond to the following questions:

1. What are your main research discipline(s) and area(s)?

**** multiple answers possible;***

**** you can chose either the overall disciplinary domain (e.g. BIOLOGY), or select one subclass / several subclasses (e.g., cellular biology, molecular biology)***

BIOLOGY

- Genetics (including epigenetics)
- Reproductive biology (e.g., biology of reproductive hormones)
- Cellular biology
- Molecular biology
- Developmental biology
- Human physiology
- Anatomy (human)
- Neuroscience (human)
- Study of human behavior, psychobiology
- Endocrinology
- Epidemiology
- Andrology
- Zoology
- Chemical Biology
- Biochemistry
- Ecology
- Evolutionary Biology
- Other (***please specify***)

MEDICINE

- Gynecology
- Obtetrics
- Paediatrics
- Pathology
- Oncology
- Clinician
- Pharmacology
- Physical medicine
- Embryologist
- Other (***please specify***)

(HUMAN) TOXICOLOGY

- Reproductive toxicology
- Computational Toxicology
- Molecular toxicology
- Carcinogenesis
- Teratogenicity
- Toxicogenomics
- Pharmacokinetics and pharmacodynamics
- Other (***please specify***)

CHEMISTRY

- environmental chemistry
- analytical chemistry
- expology (exposure to contaminants)
- Other (***please specify***)

PHARMACY

ECO-TOXICOLOGY

VETERINARY SCIENCES

STATISTICS

CHEMICAL RISK ASSESSMENT

ENVIRONMENTAL HEALTH SCIENCES

REGULATORY TOXICOLOGY

ENDOCRINE TOXICITY (TOXICITY OF ENDOCRINE DISRUPTERS)

COMPUTER SCIENCE

### bioinformatics

- Other (***please specify***)

OTHER : ***please specify***

1. Please attach your list of publications; please underline those on BPA and/or endocrine disrupters
2. Your work on BPA is:

- experimental (e.g., in laboratory)
- theoretical (e.g., expertise)
- both
- none (never worked on BPA and or endocrine disruptors)

1. Do you define yourself as having:

- a general or interdisciplinary knowledge of bisphenol A; ***please explain (one sentence)***

- a specialized knowledge of bisphenol A; ***please explain (one sentence)***

1. Expert affiliation (your main employer):

- safety agency
- academic scientists
- private scientist (industry)
- non-gouvernmental organization
- independent consultant / consultancy company

6. Do you have contracts with the industry?

- yes
- no
