# Supplementary material for: Qualichem In Vivo: A Tool for Assessing the Quality of In Vivo Studies and Its Application for Bisphenol A
Source: PLoS One. 2014 Jan 29;9(1):e87738. doi: 10.1371/journal.pone.0087738 (PMC3906223; doi:10.1371/journal.pone.0087738)
Supplement: Text S6 — Comparison between quality criteria addressed in Qualichem in vivo and quality criteria addressed in other sources. (DOC) [file pone.0087738.s006.doc]

Text S6, Qualichem in vivo: A tool for assessing the quality of in vivo studies and its application for Bisphenol A

Comparison between quality criteria addressed in Qualichem in vivo and quality criteria addressed in standardized OECD and OPPTS guidelines, in GLP guidelines, in the European quality assessments made by SCF, ECB and EFSA for Tyl et al. (2002) and Stump (2009), in the REACH on-line registration dossiers[[1]](#footnote-2) and in the ARRIVE guideline for in vivo studies.

One European quality assessment (EFSA, 2010a) presents and applies formal quality criteria for the evaluation of the study by Stump (2009). These criteria are: sufficient sample size, adequacy of control procedures, inclusion of positive controls when applicable, assessment of correlation between morphological and functional changes, and consideration of litter or dam as the appropriate statistical unit. In addition, several sources of experimental bias are considered: strain sensitivity, housing conditions, drinking bottles, phytooestrogen-containing diet, and bedding.

In the comparison matrix presented below, the empty cells correspond to Qualichem criteria that have not been dealt with at all in each of the existing practices analyzed. Blue indicates that the criterion has been briefly dealt with but without clear instructions of good practice. Green indicates that the criterion is clearly dealt with, with instructions for the experimental practice to be followed. Note that green does not mean that the criteria have been dealt with correctly (i.e., in accordance with best scientific practices) but only that they have been dealt with in detail, with clear recommendations about the experimental practice to be followed.

| ***Qualichem in vivo classes*** | ***Addressed in REACH registration (on-line version[[2]](#footnote-3))*** | ***Addressed in OECD 416 guideline*** | ***Addressed in OECD 426 guideline*** | ***Addressed in OPPTS 870.3800 guideline*** | ***Addressed in OPPTS 870.6300 guideline*** | ***Addressed in GLP*** | ***Addressed by expert committees in safety agencies*** | ***Addressed in ARRIVE[[3]](#footnote-4) guideline*** |
| --- | --- | --- | --- | --- | --- | --- | --- | --- |
| **Substance** | | | | | | | | |
| **Check of the properties of the substance and its formulations (e.g.: homogeneity, stability), before and during the experiment** | Should be documented | Demand to report the physico-chemical properties, purity and identification data for the substance, and homogeneity and stability data for formulations | Demand to report properties and purity of the substance, and concentration, homogeneity and stability of the preparation | Demand to report name, chemical abstracts service  (CAS) number or code number, strength, purity, and composition, or other appropriate characteristics. Also, stability, and if appropriate the solubility of the test, control, and reference substances under the conditions of administration |  | Chemicals should be labeled to indicate identity (with concentration) and expiry date; identity has to be verified when the substance comes from sponsor; stability and homogeneity should be known |  | Should be documented |
| **Check of the storage conditions of the substance (or of its formulations used in the experiment)** |  |  |  |  |  | Chemicals should be labeled to indicate specific storage conditions; the integrity of the chemical system should be insured; storage conditions must maintain homogeneity and stability |  |  |
| **Procedure for obtaining formulation(s) of the substance (e.g., dilution, mix with solid diet, etc.)** |  | Includes clear instructions about the good practices to follow | Demand to report details of test substance formulation/diet preparation, achieved concentration, but no indication of good practice |  |  |  |  |  |
| **Choice of the control (positive/ active or negative/ inactive)** | Should be documented | Includes clear instructions about the good practices to follow | Includes clear instructions about the good practices to follow | Includes clear instructions about the good practices to follow | Includes clear instructions about the good practices to follow |  | Used in the assessment of Stump (2009) (EFSA, 2010a)  Criterion formally established for assessing study quality: inclusion of positive controls when applicable (EFSA, 2010a) |  |
| **Experimental animals** | | | | | | | | |
| **Correspondence between the characteristics of the tested animals and the characteristics of exposed humans (e.g., age, reproductive state, etc.)** |  |  |  |  |  |  |  | Should be documented |
| **Choice of the test species / strains / sex (e.g., sensitivity)** | Should be documented | Includes clear instructions about the good practices to follow | Includes clear instructions about the good practices to follow | Includes clear instructions about the good practices to follow | Includes clear instructions about the good practices to follow | Should be documented | Criterion formally established for assessing study quality: strain sensitivity to the tested substance (EFSA, 2010a) | Should be documented |
| **Handling of experimental animals during the experiment** | Should be documented | Includes clear instructions about the good practices to follow | Includes clear instructions about the good practices to follow | Includes clear instructions about the good practices to follow | Includes clear instructions about the good practices to follow | Quarantine and follow-up of the health of animals, proper identification, cleaning and sanitation for housing and bedding |  | Should be documented |
| **Monitoring and reporting of the experimental animals’ parameters (age, weight, state of health, environmental conditions including temperature, light and humidity, etc.)** | Should be documented | Includes clear instructions about the good practices to follow | Includes clear instructions about the good practices to follow | Includes clear instructions about the good practices to follow |  |  | Criterion formally established for assessing study quality: adequacy of control procedures (but it is not specified what “control procedures” mean) (EFSA, 2010a) | Should be documented |
| **Monitoring and reporting of the state of the control group(s) at the beginning and the end of the experiment** | Should be documented | Includes clear instructions about the good practices to follow | Includes clear instructions about the good practices to follow | Includes clear instructions about the good practices to follow | Includes clear instructions about the good practices to follow |  | Criterion formally established for assessing study quality: adequacy of control procedures (but it is not specified what “control procedures” mean) (EFSA, 2010a) | Should be documented |
| **Assay** | | | | | | | | |
| **Sensitivity of the assay (ability to grasp the studied effects)** |  |  | Includes clear instructions about the good practices to follow |  | Demands to be reported, but no indications of good practice |  | Used in the assessment of Stump (2009) (EFSA, 2010a) |  |
| **Choice of the experimental unit (e.g., number of animals tested simultaneously / per group)** | Should be documented | Includes clear instructions about the good practices to follow | Includes clear instructions about the good practices to follow | Includes clear instructions about the good practices to follow | Includes clear instructions about the good practices to follow |  |  | Should be documented |
| **Number of tested groups** | Should be documented | Includes clear instructions about the good practices to follow | Includes clear instructions about the good practices to follow | Includes clear instructions about the good practices to follow | Includes clear instructions about the good practices to follow | Should be documented | Used in the assessment of Tyl et al. (2002) (SCF, 2002)  Criterion formally established for assessing study quality: sufficient sample size (EFSA, 2010a) | Should be documented |
| **Number of control groups** | Should be documented | Includes clear instructions about the good practices to follow | Includes clear instructions about the good practices to follow | Includes clear instructions about the good practices to follow | Includes clear instructions about the good practices to follow |  |  | Should be documented |
| **Scientific robustness of the regulatory guidelines (if used)** |  |  |  |  |  |  |  |  |
| **Choice to test a single substance or a mixture** |  |  |  |  |  |  |  |  |
| **Measured effects** | | | | | | | | |
| **Choice of the parameters (endpoints) for the effects to be observed** | Should be documented | Includes clear instructions about the good practices to follow | Includes clear instructions about the good practices to follow | Includes clear instructions about the good practices to follow | Includes clear instructions about the good practices to follow | Should be documented | Used in the assessment of Tyl et al. (2002) (SCF, 2002)  Used in the assessment of Stump (2009) (EFSA, 2010a) | Should be documented |
| **Choice of the observation time, duration and frequency compared to the real potential time range of the effects** | Should be documented | Includes clear instructions about the good practices to follow | Includes clear instructions about the good practices to follow | Includes clear instructions about the good practices to follow | Includes clear instructions about the good practices to follow | Should be documented |  |  |
| **Choice of the biological level observed (e.g., inter-individual, individual organism, tissue, cell, biochemical, molecular)** | Should be documented | Includes clear instructions about the good practices to follow | Includes clear instructions about the good practices to follow | Includes clear instructions about the good practices to follow | Includes clear instructions about the good practices to follow | Should be documented |  |  |
| **Precision of the effect measurement instruments and methods (e.g., visual observation, microscope, etc.)** |  | Possible measurement methods are exemplified for sperm parameters and histopathological examination only, but not for the other parameters and without indications about the good practice to follow | Bibliographic references containing observation method details are provided.  For neuropathological observations, competence of the personnel is required | Possible measurement methods are exemplified for sperm parameters, but not for the other parameters and without indications about the good practice to follow | Bibliographic references containing observation method details are provided.  Specific demands for instrument precision are provided only for measuring motor activity and for neuropathological alterations.  For neuropathological observations, competence of the personnel is required | Regular check of analytical instruments  However, the level of precision of the analytical instruments and technique, for the measured quantity, substance and matrix, is not evaluated  Details on the precision of non-instrumental methods used for recording the effects (e.g., observation) not demanded |  |  |
| **Tested exposure** | | | | | | | | |
| **Toxicokinetic stage chosen for measuring exposure (food, blood, urine, etc.)** |  |  |  |  |  |  |  |  |
| **Choice of the level of the dose tested** | Should be documented | Includes clear instructions about the good practices to follow | Includes clear instructions about the good practices to follow |  | Includes clear instructions about the good practices to follow | Should be documented |  | Should be documented |
| **Choice of the exposure duration, timing (window) and frequency compared to the real exposures** | Should be documented | Includes clear instructions about the good practices to follow | Includes clear instructions about the good practices to follow | Includes clear instructions about the good practices to follow | Includes clear instructions about the good practices to follow |  | Used in the assessment of Tyl et al. (2002) (SCF, 2002)  Used in the assessment of Stump (2009) (EFSA, 2010a) |  |
| **Choice of the number of exposure levels (doses) tested** | Should be documented | Includes clear instructions about the good practices to follow | Includes clear instructions about the good practices to follow | Includes clear instructions about the good practices to follow | Includes clear instructions about the good practices to follow | Should be documented | Used in the assessment of Tyl et al. (2002) (SCF, 2002) | Should be documented |
| **Route of administration (e.g., to animals), compared to real (e.g., humans’) routes of exposure** | Should be documented | Includes clear instructions about the good practices to follow | Includes clear instructions about the good practices to follow | Includes clear instructions about the good practices to follow | Includes clear instructions about the good practices to follow | Should be documented |  | Should be documented |
| **Precision of the exposure measurement (or analytical) instruments and methods (e.g., LD, LQ)** | Should be documented |  |  |  |  | Regular check of analytical instruments but any specification about the method (e.g., DL, QL) |  |  |
| **Control of confounders:**  **demonstration that the tested animals are really exposed to the substance of interest, to the level of interest and are not influenced by other factors (potentially influencing the effects observed)** |  | Indications are given about how to consider potential effects of the vehicle and other additives (without details), but no specification about other confounders in housing or the environment. Aspects of food and water quality should be reported, but no indication is provided about how this quality should be measured | Analysis of food and water for contaminants is demanded, but without details about the nature of these contaminants. No specification about other confounders in housing or the environment | Indications are given about how to consider potential effects of the vehicle or other additive (without details) but no specification about other confounders in housing, food, water or the environment. |  | Provisions for avoiding that handling of animals and specific care are documented (e.g., state of health, pest control treatment of bedding).  No reference to other confounders (e.g., other chemicals in tubing, housing, etc.) in GLP but regular analysis of food and water contaminants are demanded in OECD, 2000 | Criterion formally established for assessing study quality: control of confounding factors: housing conditions, drinking bottles, phytooestrogen-containing diet, bedding (EFSA, 2010a)  Water or sanitation contaminants are not considered |  |
| **Laboratory procedures and human factors** | | | | | | | | |
| **Consideration given to subjective bias:**  **minimizing experimenter’s bias through simple or/and double blinding, randomization in allocating animals to groups, inter-observer reliability** | Information provided on the respect or not of GLP | Randomization only | Randomization and blinding for observations, demand for trained personnel for neuropathological examinations |  | Randomization and blinding for observations | Qualification of laboratory personnel, standard operating procedures, study plan.  Randomization or blinding are not included |  | Should be documented |
| **Results reporting** | | | | | | | | |
| **Results reporting: right form, complete, easy to understand, reporting of the relevant experimental conditions** | Should be documented | Includes clear instructions about the good practices to follow | Includes clear instructions about the good practices to follow | Includes clear instructions about the good practices to follow | Includes clear instructions about the good practices to follow | The minimum content of the final report is specified | Used in the assessment of Tyl et al. (2002) (ECB, 2003)  Used in the assessment of Stump (2009) (EFSA, 2010a) | Should be documented |
| **Graphical representation of data and its adequacy** |  |  |  |  |  |  | Used in the assessment of Stump (EFSA, 2010a) |  |
| **The abstract is in accordance with the text of the paper** |  |  |  |  |  |  |  | Should be documented |
| **Results analysis** | | | | | | | | |
| **Choice of the statistical method for analyzing the study results** | Should be documented | General indications but no specific rule of good practice | General indications but no specific rule of good practice | General indications but no specific rule of good practice |  | Should be documented |  | Should be documented |
| **Choice of the statistical unit** | Not explicitly demanded, available only if the registrant includes it voluntarily |  | Includes clear instructions about the good practices to follow |  |  |  | Criterion formally established for assessing study quality: consideration of litter or dam as the appropriate statistical unit (EFSA, 2010a) | Should be documented |
| **Treatment of data before statistical analysis** | Not explicitly demanded, available only if the registrant includes it voluntarily |  | Should be discussed |  |  |  | Used in the assessment of Stump (2009) (EFSA, 2010a) |  |
| **Statistical power** | Not explicitly demanded, available only if the registrant includes it voluntarily |  |  |  |  |  |  |  |
| **Analysis of errors, uncertainty and of study limitations** |  |  |  | A description of all circumstances that may have affected the quality or integrity of the data is demanded |  |  |  | Should be documented |
| **Causal interpretations** | | | | | | | | |
| **Interpretation of the dose – response relationship** |  | Demand to evaluate the causal relationship but without good practices to follow | Includes clear instructions about the good practices to follow | Demand to evaluate the relationship between exposure and effects, but without good practices to follow | Includes clear instructions about the good practices to follow |  | Used in the assessment of Tyl et al. (2002) (ECB, 2003)  Criterion used for assessing studies but not explicitly included in the list of formal quality criteria: clear dose response curves (EFSA, 2010a) |  |
| **Interpretation of the biological mechanism / biological significance of the findings** |  |  | Should be discussed |  |  |  | Criterion used for assessing studies but not explicitly included in the list of formal quality criteria: lack of a common clearly defined mode of action of BPA at low doses (EFSA, 2010a) |  |
| **Interpretation of the relevance of animal data for humans** |  | Includes clear instructions about the good practices to follow |  |  |  |  |  | Should be documented |
| **Interpretation of the functional relevance (as an “effect”) of behavioral, morphological, histological, molecular or biochemical changes** |  |  | Should be discussed |  | Should be discussed, but no further indication of good practice is given |  | Criterion formally established for assessing study quality: assessment of correlation between morphological and functional changes (EFSA, 2010a) |  |
| **Results interpretation: epistemological** | | | | | | | | |
| **General level of theoretical understanding of the substance, its fate in the body, its biological effects, its relevant biological mechanisms of action of generally of its toxicology** |  |  |  |  |  |  | Used in the assessment of Stump (2009) (EFSA, 2010a) |  |
| **Results check** | | | | | | | | |
| **Status of peer-review** |  |  |  |  |  | External inspections by empowered control organisms and internal quality assurance insure compliance with GLP |  |  |
| **Coherence with other studies** |  | Includes clear instructions about the good practices to follow | Includes clear instructions about the good practices to follow |  |  |  | Used in the assessment of Stump (2009) (EFSA, 2010a) | Should be documented |
| **Results interpretation: expert judgment** | | | | | | | | |
| **Concordance between the interpretation of the results (i.e., in terms of level of evidence and conclusiveness) and the raw data** |  |  | Includes clear instructions about the good practices to follow |  | Use of professional judgment must be reported and argumented |  | Used in the assessment of Stump (2009) (EFSA, 2010a) |  |
| **Analysis of assumptions (e.g., that replace missing knowledge in toxicology, missing data, etc.)** |  |  |  |  |  |  |  |  |
| **Variability** | | | | | | | | |
| **Reporting and analysis of natural / unexplained variability** |  |  |  |  | Reporting of standard deviation for each continuous endpoint is demanded |  | Used in the assessment of Stump (2009) (EFSA, 2010a) |  |

1. The Stump study : [http://apps.echa.europa.eu/registered/data/dossiers/DISS-9dbe071c-c12d-0fe1-e044-00144f67d249/AGGR-5141f264-231c-4514-b8a9-4d4ecfb3527c_DISS-9dbe071c-c12d-0fe1-e044-00144f67d249.html#AGGR-5141f264-231c-4514-b8a9-4d4ecfb3527c](http://apps.echa.europa.eu/registered/data/dossiers/DISS-9dbe071c-c12d-0fe1-e044-00144f67d249/AGGR-5141f264-231c-4514-b8a9-4d4ecfb3527c_DISS-9dbe071c-c12d-0fe1-e044-00144f67d249.html#AGGR-5141f264-231c-4514-b8a9-4d4ecfb3527c)

   The Tyl study: [http://apps.echa.europa.eu/registered/data/dossiers/DISS-9dbe071c-c12d-0fe1-e044-00144f67d249/AGGR-f07e3c0e-5b3f-4698-b36c-a2a42b97529c_DISS-9dbe071c-c12d-0fe1-e044-00144f67d249.html#AGGR-f07e3c0e-5b3f-4698-b36c-a2a42b97529c](http://apps.echa.europa.eu/registered/data/dossiers/DISS-9dbe071c-c12d-0fe1-e044-00144f67d249/AGGR-f07e3c0e-5b3f-4698-b36c-a2a42b97529c_DISS-9dbe071c-c12d-0fe1-e044-00144f67d249.html#AGGR-f07e3c0e-5b3f-4698-b36c-a2a42b97529c) [↑](#footnote-ref-2)
2. URL for BPA : <http://apps.echa.europa.eu/registered/data/dossiers/DISS-9dbe071c-c12d-0fe1-e044-00144f67d249/DISS-9dbe071c-c12d-0fe1-e044-00144f67d249_DISS-9dbe071c-c12d-0fe1-e044-00144f67d249.html> [↑](#footnote-ref-3)
3. In addition, ARRIVE includes: title, background, objectives, ethical statement and funding sources. [↑](#footnote-ref-4)
