# Supplementary material for: Qualichem In Vivo: A Tool for Assessing the Quality of In Vivo Studies and Its Application for Bisphenol A
Source: PLoS One. 2014 Jan 29;9(1):e87738. doi: 10.1371/journal.pone.0087738 (PMC3906223; doi:10.1371/journal.pone.0087738)
Supplement: Text S7 — Scale for quality assessment. (DOC) [file pone.0087738.s007.doc]

Text S7, Qualichem in vivo: A tool for assessing the quality of in vivo studies and its application for Bisphenol A

**Scale for quality assessment**

For assessing the quality of each criterion, please respond to the question, using one of the following eight answers:

| **Answer** | **On a scale from 1 to 6, the answer corresponds to the score** |
| --- | --- |
| Agree strongly | 6 |
| Agree moderately | 5 |
| Agree slightly | 4 |
| Disagree slightly | 3 |
| Disagree moderately | 2 |
| Disagree strongly | 1 |
| I cannot answer | CA |
| Not applicable | NA |

Please say **WHY** you have provided this score:

1. This is my intimate conviction
2. I believe there is ONE reason:

- …………….

1. I believe there are TWO reasons:

- …………..
- …………..

1. I believe there are THREE or MORE reasons:

- ……………….
- ……………….
- ……………….
- ……………….
- ……………….

*Note: Qualichem could be improved by adding a requirement that respondents provide scientific references from the peer-reviewed literature to support their choice of scores.*
